# GATA-1 Inhibits *PU*.*1* Gene via DNA and Histone H3K9 Methylation of Its Distal Enhancer in Erythroleukemia

**DOI:** 10.1371/journal.pone.0152234

**Published:** 2016-03-24

**Authors:** Pavel Burda, Jarmila Vargova, Nikola Curik, Cyril Salek, Giorgio Lucio Papadopoulos, John Strouboulis, Tomas Stopka

**Affiliations:** 1 Biocev and Pathological Physiology, 1st Faculty of Medicine, Charles University in Prague, Czech Republic; 2 Institute of Hematology and Blood Transfusion, Prague, Czech Republic; 3 Institute of Molecular Biology and Biotechnology, Foundation of Research and Technology-Hellas, Heraklion, Crete, Greece; 4 Department of Biology, University of Crete, Heraklion, Crete, Greece; 5 1st Medical Department–Hematology, General Faculty Hospital, Prague, Czech Republic; Queen's University Belfast, UNITED KINGDOM

## Abstract

GATA-1 and PU.1 are two important hematopoietic transcription factors that mutually inhibit each other in progenitor cells to guide entrance into the erythroid or myeloid lineage, respectively. PU.1 controls its own expression during myelopoiesis by binding to the distal URE enhancer, whose deletion leads to acute myeloid leukemia (AML). We herein present evidence that GATA-1 binds to the *PU*.*1* gene and inhibits its expression in human AML-erythroleukemias (EL). Furthermore, GATA-1 together with DNA methyl Transferase I (DNMT1) mediate repression of the *PU*.*1* gene through the URE. Repression of the *PU*.*1* gene involves both DNA methylation at the URE and its histone H3 lysine-K9 methylation and deacetylation as well as the H3K27 methylation at additional DNA elements and the promoter. The GATA-1-mediated inhibition of *PU*.*1* gene transcription in human AML-EL mediated through the URE represents important mechanism that contributes to PU.1 downregulation and leukemogenesis that is sensitive to DNA demethylation therapy.

## Introduction

Hematopoietic differentiation is controlled by the interplay of opposing lineage-specific transcription factors: PU.1 and GATA-1. If one of these factors predominates it ultimately leads to a specification of a particular lineage [1–5]. PU.1 binds the URE and auto-regulates *PU*.*1* gene expression [6] to achieve the different levels of PU.1 that regulate differentially monocytic, granulocytic, or lymphocytic programs [7, 8]. While deletion of the *PU*.*1* gene completely abrogates myeloid and/or lymphoid development in mice, transgenic disruption of the upstream regulatory element (URE) results in ~80% reduction of PU.1 level and AML [1, 9]. Disruption of the URE by the integrated spleen focus forming provirus (SFFV) associates with the development of AML-EL [10, 11]. Mutations of *PU*.*1* gene in human AML are sporadic and therefore the epigenetic mechanisms regulating PU.1 level may be more important.

GATA-1 is a lineage specific factor that regulates erythroid, megakaryocytic, mast cell and eosinophil differentiation [12–16] while it inhibits myeloid genes that are controlled by PU.1 [17, 18]. GATA-1 transcriptionally inhibits the *PU*.*1* gene in murine erythroblasts through its promoter [19]. However, most of the *PU*.*1* gene transcription is regulated by the DNA elements [20] located upstream of the promoter. We herein have elucidated that GATA-1 represses the *PU*.*1* gene through these DNA elements by binding to the URE and associating with DNMT1 leading to DNA methylation and H3K9-trimethylation. However, this mechanism is not capable completely silencing the PU.1 expression in AML-EL, thus counteracting the blockade of erythroid differentiation.

## Materials and Methods

### Chromatin Immunoprecipitation (ChIP) and mRNA/Protein Expression

OCI-M2, SKM1, HeLa (DSMZ, Germany), and K562 (ATCC, UK) cells were cultured in Iscove’s Dulbecco’s medium supplemented with FBS/antibiotics. Primary cells: Written donor’s informed consent (based on 1964 Declaration of Helsinki) was obtained. The Ethical Committee of the Institute of Hematology and Blood Transfusion (Prague) approved this study. AML-EL CD34+ cells were magnetically sorted with 95% purity. Normal CD34+ cells were obtained from Lonza (clones 8&9). ChIP: 10^7^cells were cross-linked in 1% formaldehyde, lysed and sonicated [21, 22]. IP-antibodies: GATA-1 (N6/sc265-Santa Cruz, USA), PU.1 (sc352-Santa Cruz, USA), DNMT1 (Ab13537-Abcam, UK), H3K9Ac (07-352-Upstate, USA), H3K9Me3 (Ab88-98-Abcam, UK), H3K4Me3 (pAb003-050-Diagenode, Belgium), H3K27Me3 (Ab6002-Abcam, UK), and control antibody (NI01-EMB Biosciences, USA). Enrichment quantitation and student T-test for a set of biological replicates (duplicates or triplicates) was done as published previously [23]. A one-way ANOVA followed by Tukey’s HSD was performed to compare multiple groups. RT-PCR: total RNA was isolated (RiboZol, Amresco, Solon, OH, USA), reverse-transcribed and PCR-amplified (9700HT Instrument). Immunoblotting: ~3x10^7^ cells were lysed with RIPA buffer and gently sonicated. 20μg of the protein lysates were resolved on a 4–12% gradient Bis-Tris gel (NuPage-Life Technologies, USA) and dry-blotted (iBlot-Life technologies, USA). WB^primary^: anti-PU.1 (T-21/sc-352), Anti-β-actin (I-19/sc-1616; Santa Cruz, CA, USA). Co-IP^primary^: anti-DNMT1 (Ab13537-Abcam, UK), anti-GATA-1 (sc265) and anti-PU.1 (sc352, both Santa Cruz, USA), control IgG (NI01-EMB Biosciences, USA). After a 2h-incubation, the complexes were washed 3x in buffer (0.1%Triton X-100 50mM Tris-HCl (pH7.4), 300mM NaCl, 5mM EDTA), resolved by SDS/PAGE, blotted, and immunodetected. Double-staining immunofluorescence: primary anti-GATA-1 (4F5/Ab98953-Abcam, UK), anti-PU.1 (9G7/2258-Cell Signaling Technology), and AlexaFluor488 and AlexaFluor594 (Thermo Fischer Scientific, USA) were used with Vectashield Mounting Medium containing DAPI and detected by Leica TCS-SP2+AOBS system.

### Reporter Gene Assay and DNA Methylation

hGATA-1 siRNA (Santa Cruz Biotechnology, Dallas, TX, USA, sc-29330) or negative control oligo (sc-37007), or mGATA-1 cDNA expression plasmid (pXM-GATA-1 [24]; not recognized by human primers) were electroporated by Amaxa (VPA-1001 or VCA-1003) each with the GFP-expressing vector pMaxGFP. HeLa cells were transfected using Lipofectamine 2000 reagent (Thermo Fischer Scientific, Life technologies, USA). Transfection efficiency was monitored by flow cytometry analysis. The cells were alternatively transfected also with 2μg of pGL3 vector and equimolar amounts of the PU.1 reporter constructs and analyzed by a Dual Luciferase Assay (E1910, Promega, USA). DNA methylation: bisulfite-treated DNA (EpiTect bisulfite kit) was amplified/purified by a QIAquick gel extraction kit (Qiagen, Venlo, Netherlands), subcloned into a pCR 2.1-TOPO-vector, and sequenced using a BigDye terminator chemistry (Thermo Fischer Scientific, Life technologies, USA).

## Results

### 1) GATA-1 Represses the *PU*.*1* Gene and Blocks Proliferation in Human EL

Unlike most AMLs, the AML-ELs co-express erythroid and myeloid cell markers. We herein utilized two unrelated human erythroleukemic cell lines (OCI-M2 and K562) and primary human AML-EL CD34+ progenitors that all expressed GATA-1 and PU.1 mRNAs. Non-hematopoietic HeLa cells as expected did not express GATA-1 or PU.1 (S1A Fig). The Western blots (S1B Fig) and immunofluorescence (S2A Fig) confirmed the expression of GATA-1 and PU.1 in the AML-ELs. Furthermore, immunofluorescence shows a partial overlap between GATA-1 and PU.1 signals (S2B Fig). Thus GATA-1 and PU.1 are co-expressed at similar level in the human AML-EL.

**Fig 1:**
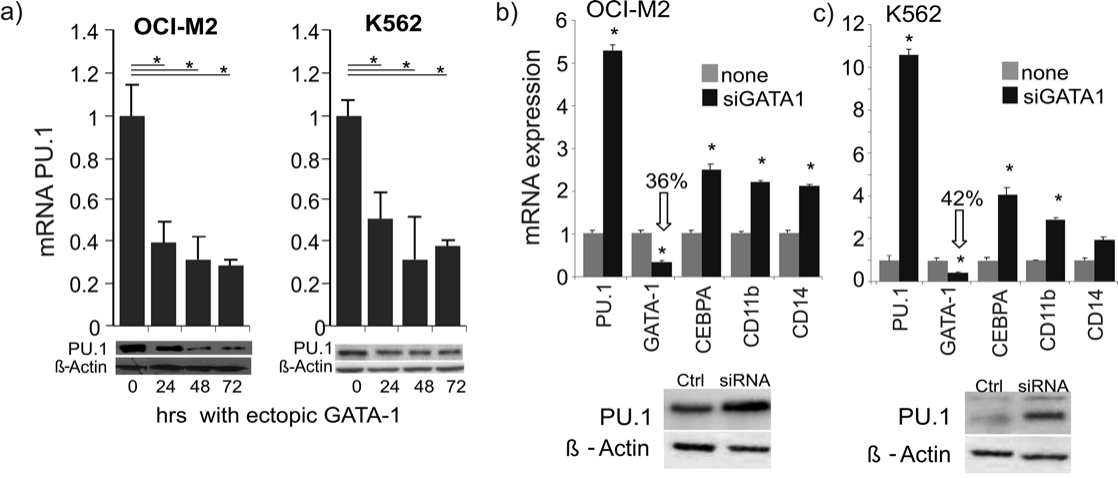
Ectopic GATA-1 inhibits PU.1 expression and GATA-1 knockdown stimulates PU.1 expression in AML-ELs. (a) mRNA level of PU.1 following pXM-GATA-1 transfection into OCI-M2 (left) and K562 (right) cells, 0 to 72hrs. Data from two independent experiments that were carried out in duplicate are shown. Immunoblots of PU.1 and beta-actin are shown below the graphs. (b,c) Anti-GATA-1 siRNA oligo or scrambled control oligo were transfected into OCI-M2 (b, 20nm) and K562 (c, 40nM) cells. Total mRNA was purified and subjected to quantitative reverse transcription-PCR (TaqMan) at 48hrs after siRNA transfection. mRNA expression (of the genes indicated on the X axis) is calculated relative to negative control siRNA transfections and to HPRT mRNA levels. The arrows with numbers indicate the fold decreases of GATA-1 level after siRNA inhibition. Immunoblotting for PU.1 and ß-Actin in OCI-M2 and K562 cells upon GATA-1 siRNA transfection shown below.

We next tested whether *PU*.*1* gene expression can be affected by GATA-1 and therefore we upregulated GATA-1 by transfecting a GATA-1-expressing plasmid (pXMGATA-1) into AML-EL cells (OCI-M2, K562) and monitored the levels of PU.1 mRNA and protein for 72hrs. Upon GATA-1 transfection we observed repression of PU.1 whose mRNA decreased below 30%, with a major decrease seen within the first 24hrs (Fig 1A). Similarly to mRNA, PU.1 protein levels were downregulated as evidenced by immunoblotting (lower panels in Fig 1A). It is known that PU.1 regulates several intermediate target genes, including transcription factor CEBPA and surface markers (CD14, CD11b) [25]. Indeed, GATA-1-mediated repression of PU.1 was followed by the downregulation of these targets (S3A and S3B Fig). These data show that in the cells transfected by murine Gata1-encoding expression plasmid the GATA-1 exerts a repressive role on *PU*.*1* gene expression, leading to further inhibition of PU.1-dependent genes. We also noted that transfection of pXMGATA-1 into AML-ELs resulted in the inhibition of the proliferation coincidently with the decrease of mRNA expression of myeloid lineage markers (S3 Fig). Increased GATA-1 expression in the MEL cells was previously shown to induce cell cycle arrest, together with partial differentiation induction [22]. Taken together, GATA-1 overexpression in AML-ELs downregulated PU.1, as well as its targets, thus supporting our hypothesis that GATA-1 could be a repressor of the *PU*.*1* gene.

### 2) GATA-1 Knockdown Upregulates PU.1 Levels in Human AML-EL

As a next step, we tested the role of GATA-1 as a *PU*.*1* gene repressor by inhibiting GATA-1 with specific siRNAs, previously shown elsewhere to be effective [26]. Titration of siRNAs identified the most efficient concentration and timing, which resulted in significant downregulation of GATA-1. Expression of PU.1 and its targets were monitored for 48hrs after addition of siRNA. Knockdown of GATA-1 blocked its mRNA expression ~3-fold, resulting in ~5-fold upregulation of PU.1 mRNA in AML-EL (OCI-M2, Fig 1B). Similar results were noted for GATA-1 knockdown in another AML-EL (K562, ~2-fold and ~10-fold respectively) (Fig 1C). Upregulation of PU.1 mRNA was again coincident with upregulation of PU.1 target genes: CEBPA, CD11b, and CD14 mRNAs (Fig 1B and 1C). GATA-1-mediated derepression of PU.1 was also noted at the protein level (Fig 1B and 1C). These experiments again supported the notion that GATA-1 levels are sensed at the *PU*.*1* gene which responds by its upregulation upon decrease of the putative repressor GATA-1.

### 3) GATA-1 Binds to the PU.1 Locus in Human EL

The PU.1 upregulation observed as early as at 24hrs and reaching maximum at 48hrs upon inhibition of GATA-1 (and conversely PU.1 downregulation upon increasing GATA-1 levels), as well as the dynamic changes at both mRNA and protein levels suggested that GATA-1 transcriptionally inhibits *PU*.*1* gene. To further test this we asked whether GATA-1 binds to the PU.1 locus. Firstly, using the Genomatix Gene Analyzer (www.genomatix.de) we visualized 59 predicted GATA-1 binding T/A(GATA)A/G DNA motifs and 23 PU.1 GAGGAA motifs within an 18kb region upstream from the TSS. The PU.1 motifs were taken into account because we have previously suggested that GATA-1 could access DNA through an association with PU.1 [22]. Next, we assayed these predicted binding sites using qChIP in human AML-ELs and used the myelo-monocytic AML (not expressing GATA-1) and HeLa cells as controls. The chromatin was sonicated to minimize proportional presence of fragments larger than 500bp. PCR primers were designed to cover all 82 predicted GATA-1/PU.1 DNA binding sites. The amplicons covered important regions such as the URE (-17.5,-16.5kb), the -13.4kb enhancer (E) and the proximal promoter (PP).

**Fig 2:**
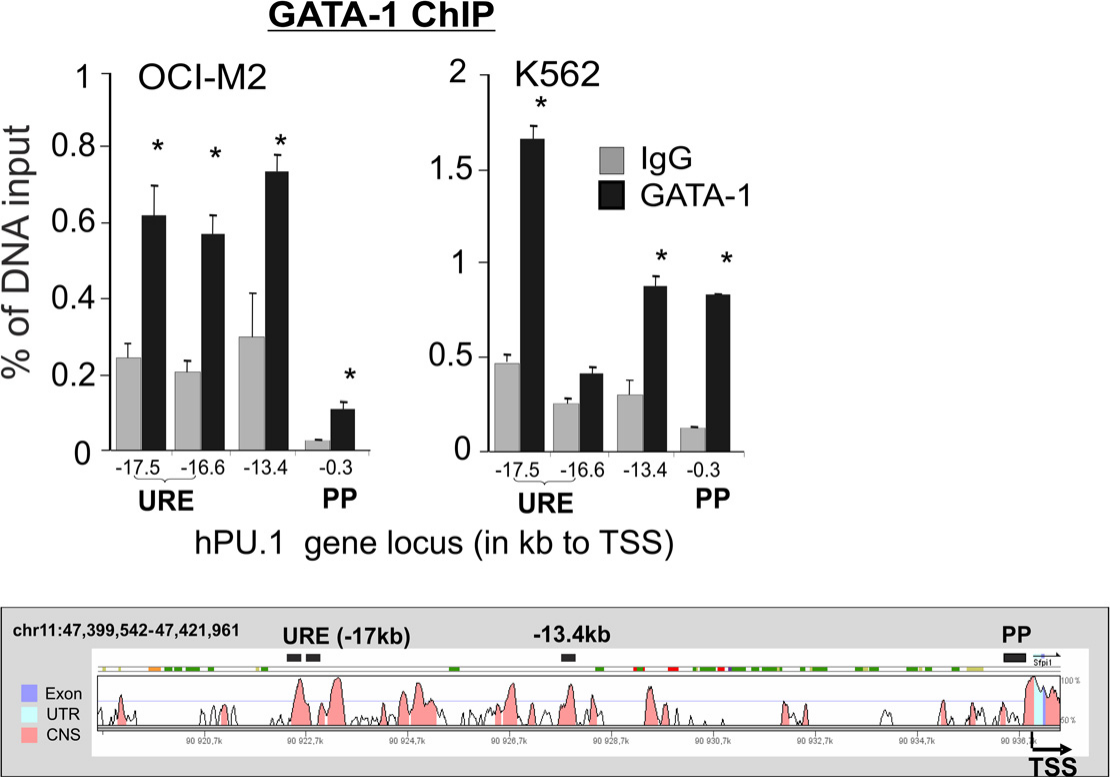
GATA-1 occupies the *PU*.*1* gene locus in AML-ELs. GATA-1 ChIP in OCI-M2 (left) and K562 (right). PCR amplicons positions are relative to TSS (kb). Specific signals are expressed as % of DNA input. Nonspecific signals of IgG immunoprecipitates are shown as gray columns. Two independent experiments were carried out in duplicate. Error bars: SE, *p≤0.05. Bottom: Vista plot of the *PU*.*1* gene with indicated positions of PCR amplicons. URE (upstream regulatory element), PP (proximal promoter region).

Significant GATA-1 occupancy was observed at the URE, at the -13.4E and also with slightly lower signal at PP in both AML-ELs (Fig 2 and S4 Fig). Significant co-occupancy with PU.1 was noted at the URE (S5A Fig) where no GATA-1 binding sites were predicted, suggesting that GATA-1 is bound through PU.1 previously established to bind to the URE [20]. This is observable also in the control SKM1 cell line that displays PU.1 binding at the URE in the absence of GATA-1 expression (S5B Fig). Interestingly, within the PU.1 upstream locus there also exist regions including PP and -13.4E that are not occupied by PU.1 but have detectable occupancy by GATA-1. The signals appear quite specific, as no such signals were detected in the HeLa control and all signals were compared to immunoprecipitations with control non-binding antibodies. These experiments confirmed that in AML-ELs GATA-1 could bind to the *PU*.*1* gene locus. The GATA-1 binding pattern partially overlaps with PU.1 occupancy, especially at the URE whilst at other important regions, such as the -13.4E and the PP, the binding of GATA-1 does not overlap with PU.1 binding. Collectively, these data implicate GATA-1 as being a putative repressor of the *PU*.*1* gene through interactions with the URE,-13.4E and PP.

### 4) GATA-1 Transcriptionally Inhibits *PU*.*1* Gene in Human ELs

**Fig 3:**
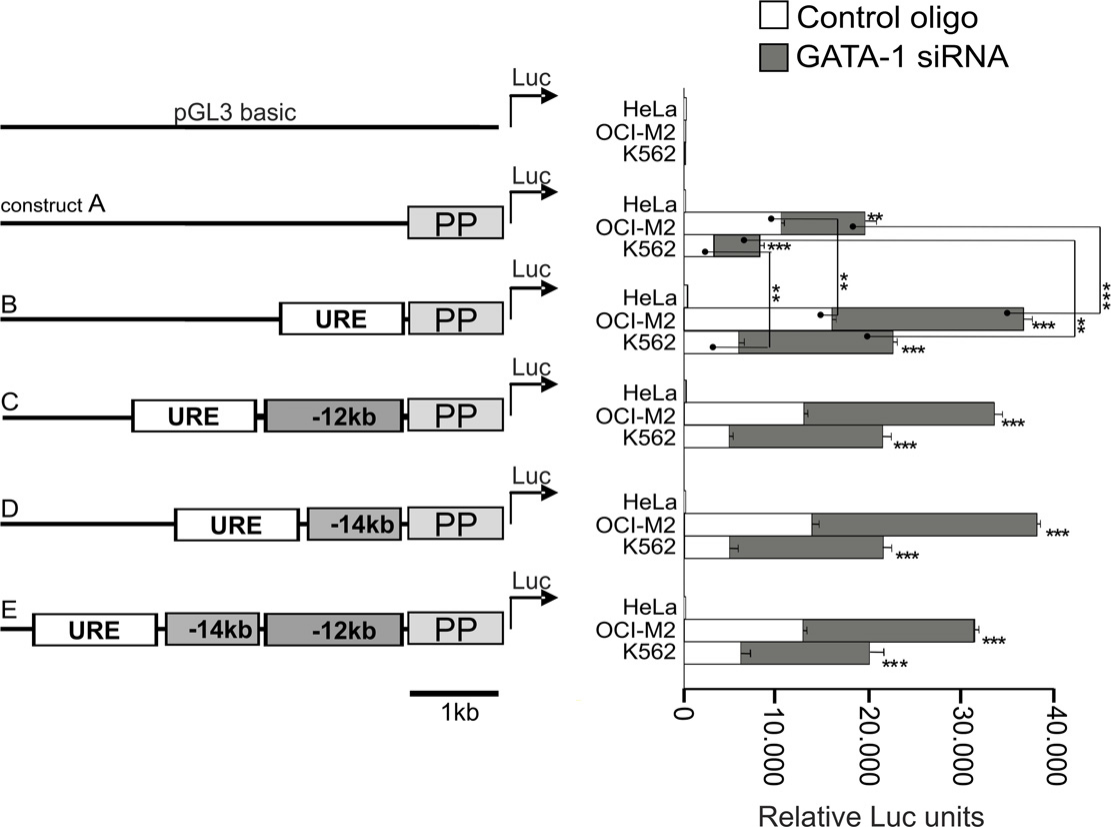
Reporter gene assays showing that specific PU.1 element/s are repressed by GATA-1 in AML-ELs. The pGL3 basic plasmid was linked to the following upstream PU.1 elements: PP = proximal promoter, -12kbE, -14kbE, and the URE (or different combinations thereof, reporter constructs are named A-E were transfected into OCI-M2 and K562 cells either with scrambled control oligo (white bars) or with GATA-1 siRNA oligos (grey bars). HeLa cells served as control. Luciferase activity is normalized to the amount of proteins in each sample. Asterisks tightly to grey bars mark significances between signals from Control oligo and siRNA GATA-1 transfection within one cell line and one type of reporter. Complete ANOVA analysis results between all relevant transfections are displayed in table in S3 Fig. Marks of significance: *p < 0.05, **p < 0.01, ***p < 0.001.

We next tested what *PU*.*1* gene regions mediate the GATA-1 repressive signals using a transcriptional reporter assay. We utilized four plasmids with different portions of the *PU*.*1* upstream region (URE, -13.4E and -12E) all attached to the PP linked to a luciferase reporter gene (Fig 3). The reporter constructs did not express any luciferase when transfected into the control HeLa cells. Next, the constructs were transfected into the AML-ELs resulting into a signal gain that was approximately 10–20 thousand RLU. This allowed us to perform the experiment (similar to that in Fig 1B) utilizing GATA-1 knockdown to test whether GATA-1 blocks luciferase gene activation imposed on the PU.1 reporter vector and to find what portions of *PU*.*1* gene are necessary for this process. The knockdown was significant, resulting in a reduction to approximately 30% of GATA-1 levels. Our data from the reporter assay show that the PP mediates some of the GATA-1 mediated repression of the *PU*.*1* gene. However, the URE adds significantly more to this process and mediated repression of *PU*.*1* as evidenced upon GATA-1 knockdown (Fig 3). Addition of -13.4E did not enhance the derepression, indicating that GATA-1 does not mediate any additional repression through this region. In addition, we also utilized another human SKM1 cell line that expresses PU.1 and not GATA1 and confirmed that the GATA-1 siRNA have no off target effects on the reporter constructs containing PP or URE (S6B Fig). Thus, GATA-1 mediated repression of the *PU*.*1* gene is mediated mostly through the URE and PP.

### 5) GATA-1-Imposed Histone Modifications at the PU.1 Locus in the hAML-ELs

We have previously shown that ectopic GATA-1 expression inhibits PU.1 target genes [22] and modulates levels of H3K9 acetylation that are considered to represent an active chromatin mark. We asked whether the GATA-1 mediated repression involves any of the established chromatin marks, such as H3K9Me3, H3K27Me3 (marks of repressed chromatin) and also H3K9Ac at the *PU*.*1* gene locus in AML-ELs (OCI-M2,K562) and the control cell lines (SKM1,HeLa), all transfected with pXMGATA-1.

**Fig 4:**
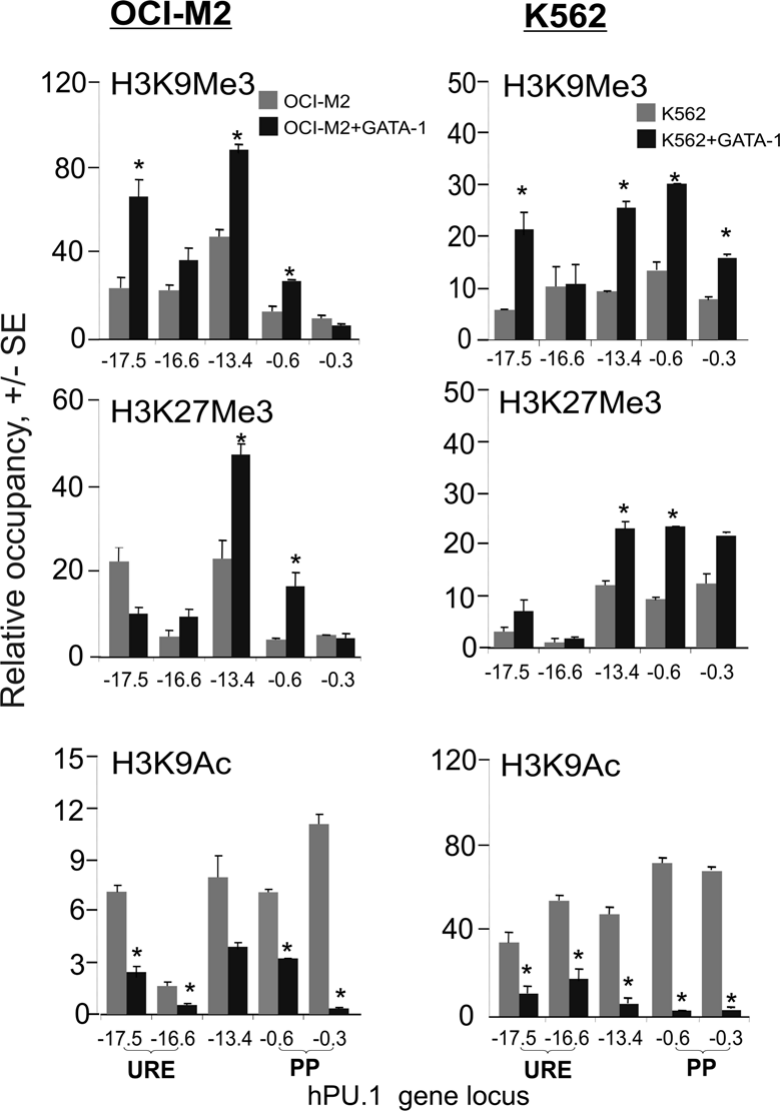
Repressive histone modifications following GATA-1 overexpression in AML-ELs. ChIP at the *PU*.*1* gene locus was carried out for the H3K9Me3, H3K27Me3, and H3K9Ac histone tail modifications in OCI-M2 (left) and K562 (right) cells. Grey bars: control cells, dark bars: 48hrs after GATA-1 transgene transfection. Data are relative to control antibody IPs (Y axis). T-test significance: p<0.05 (star). Amplicon positions are shown on the X axis.

Firstly, the repressive mark H3K9Me3 was enriched (~10-30fold) and significantly enhanced (~3-4fold) by GATA-1 overexpression, and also overlapped with GATA-1 occupancy (see Fig 4^upper panels^ and S7B Fig) at the URE, the -13.4E and near the PP in the AML-ELs, whereas this was not observed in the SKM1 or HeLa cells (S7A Fig). Next, the H3K27Me3 mark was enriched (~5–20 fold) and significantly enhanced (~2fold) by GATA-1 overexpression, and also overlapped with GATA-1 occupancy (see Fig 4 middle panels) at the -13.4E and PP. Lastly, the H3K9Ac mark was detectable at the PU.1 locus in the AML-ELs (and also in the SKM1 cells but not in HeLa, S7A Fig). Following the overexpression of GATA-1, the H3K9Ac mark was significantly decreased at multiple amplicons upstream of the *PU*.*1* gene. Taken together, both repressive marks H3K9Me3 and H3K27Me3 are increasingly detectable in the GATA-1-bound regions of the *PU*.*1* gene. While H3K9Me3 is enriched also in the URE and was previously linked to PU.1/GATA-1 interactions on DNA, the second repressive modification H3K27Me3 was also detected at the regions that are occupied by GATA-1 but not by PU.1 (-13.4E and PP, see S7B Fig). H3K9 acetylation at the PU.1 locus is rather broad and its decrease upon GATA-1 overexpression in the AML-ELs was noted at multiple PU.1 enhancers. Taken together, this experiment supports the role of GATA-1 as a repressor of the *PU*.*1* gene, involving modification of chromatin such as histone H3K9/H3K27 hyper(tri)methylation and H3K9 hypoacetylation.

### 6) GATA-1 and PU.1 Occupy Upstream Enhancers but Not URE in Murine EL

**Fig 5:**
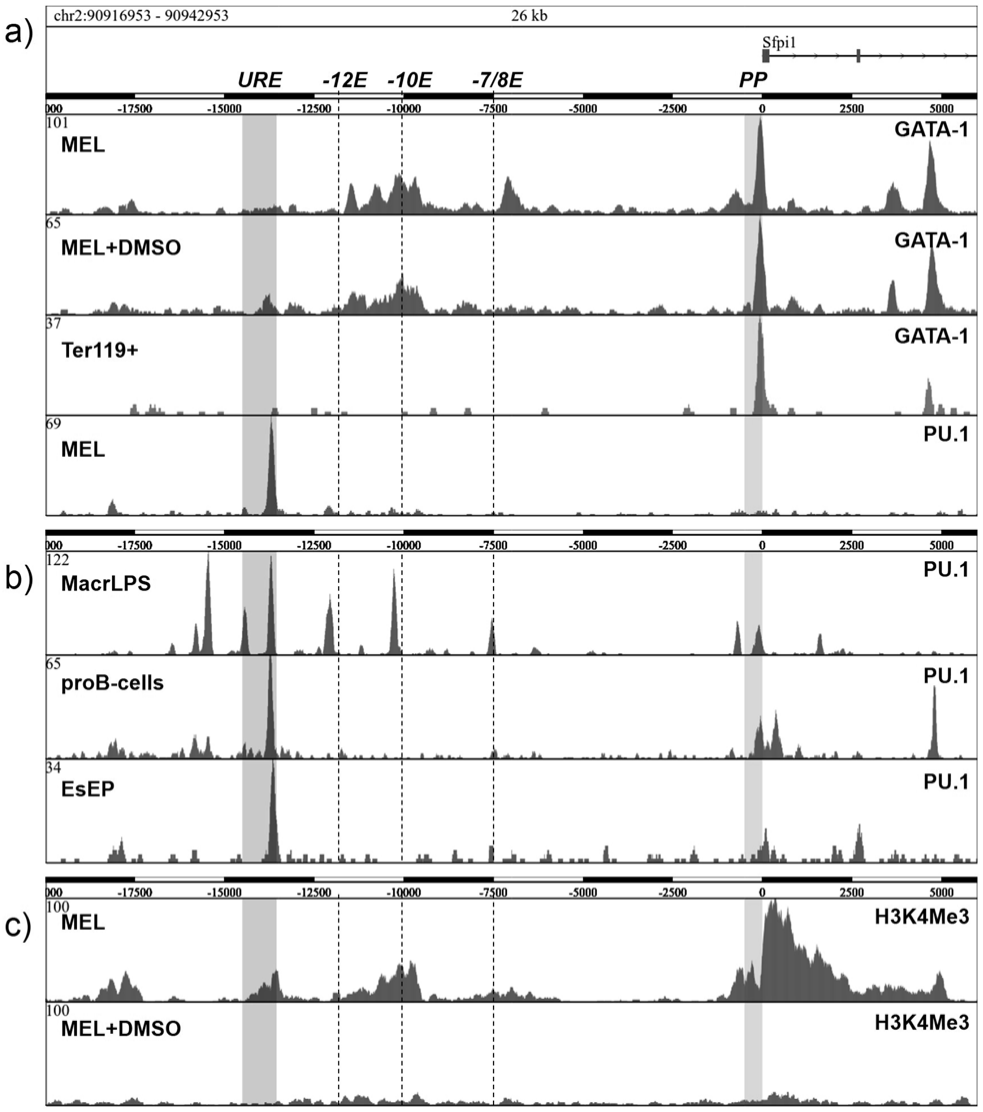
ChIP-seq read density profiles of GATA-1, PU.1 and H3K4me3 datasets at the *PU*.*1* (*Sfpi1*) gene locus (-20kb upstream to 6kb downstream of PU.1 TSS). The proximal promoter (PP, 0 to 500nt upstream the TSS), the URE (-13548bp; -14505bp) and conserved regions corresponding to the human genome are highlighted in vertical stripes and lines. (A) Occupancy of GATA-1 in MEL cells or MEL induce to differentiate by 2% DMSO for 5 days, or in Ter119+ Fetal liver (E14.5-derived) erythroblasts. The lowest panel shows occupancy by PU.1 in MEL cells. (B) Binding profiles of PU.1 and GATA1 in murine primary macrophages stimulated by lipopolysaccharide for 24hrs (MacrLPS), pro-B cells (38B9), and EsEP: differentiating murine ES cell-derived erythroid progenitors; (C) H3K4me3 occupancy in MEL and MEL+DMSO cells.

The murine (m) and human (h) *PU*.*1* gene sequences share significant homologies that also include the PU.1 enhancers. We asked whether GATA-1 similarly represses the *PU*.*1* gene in the mELs, as it does in hELs. We utilized ChIP-seq data from mEL (MEL) cells to address this question. MEL cells display the transcriptional interference of the PU.1 expression imposed by the integrated SFFV provirus at mURE [1]. Unlike the PU.1^URE/URE^ model, MEL cells are blocked at a later stage of erythroid development as they contain a mixture of rapidly proliferating proerythroblasts and late basophilic erythroblasts. MEL cells can be differentiated upon induction with activated GATA-1 or with chemical inducers (DMSO, HMBA) into orthochromatophillic erythroblasts and this is coupled with downregulation of PU.1 [27]. ChIP in MEL cells demonstrated the GATA-1 occupancy (similarly to hAML-ELs) in the PU.1 promoter region, together with a relatively broad occupancy at positions -7/8kb, -10kb, and -12kb (see Fig 5A; note that the localization of murine enhancers have different coordinates compared to human enhancers). Upon DMSO-induced differentiation, the occupancy of GATA-1 was similar except that it decreased in the -7/8kb Element. By contrast, in physiological fetal liver-derived erythroblasts that have already underwent PU.1 repression, GATA-1 occupancy of the *PU*.*1* gene locus is found only at the promoter but not at other regions (Fig 5A), indicating that it is at a relatively later stage of erythroid differentiation. Occupancy of PU.1 in proliferating MEL cells was found only at the URE (Fig 5A). However, in myelo-lymphoid cells the PU.1 occupancy is much broader, for example, in macrophages 9 different regions are occupied by PU.1, or in pro-B cells PU.1 occupancy is seen at the URE and the promoter. Similarly to MEL cells, PU.1 occupancy in ES-derived erythroid progenitor cells is found only at the URE (see ES derived progenitors, Fig 5B). We have also assessed histone-tail modifications across the *PU*.*1* gene locus in the mAML-EL context. Whereas H3K9Me3 and H3K27Me3 were not highly enriched at the *PU*.*1* gene locus (S8 Fig), we focused on the H3K4Me3 mark, which was enriched in MEL cells at the PU.1 promoter and at several enhancers including the URE. The H3K4Me3 mark, which represents an active chromatin mark, associated with RNA Pol-II transcription, was significantly decreased at all PU.1 regions upon DMSO-induced MEL differentiation (Fig 5C), suggesting that it is associated with *PU*.*1* gene repression. MEL cells unlike the hAML-ELs are characterized by occupancy of GATA-1 at different positions within the PU.1 locus but also include those that we detected in hELs, such as the promoter and -13.4kb enhancer. However, the GATA-1 occupancy (and H3K9 and H3K27 trimethylation) at the URE was not observed in MEL cells but its negligible traces appeared there upon silencing of the PU.1 expression by DMSO. Furthermore, GATA-1 occupancy in MEL cells was overlapped by H3K4Me3 (that decreased upon PU.1 silencing) but did not overlap with modifications of the H3K9 or H3K27 residues. Taken together, the repression of the PU.1 gene by GATA-1 in murine context is entirely different as compared to human AML-EL.

### 7) GATA-1 Associates with DNMT1 to Methylate the URE in the AML-EL

**Fig 6:**
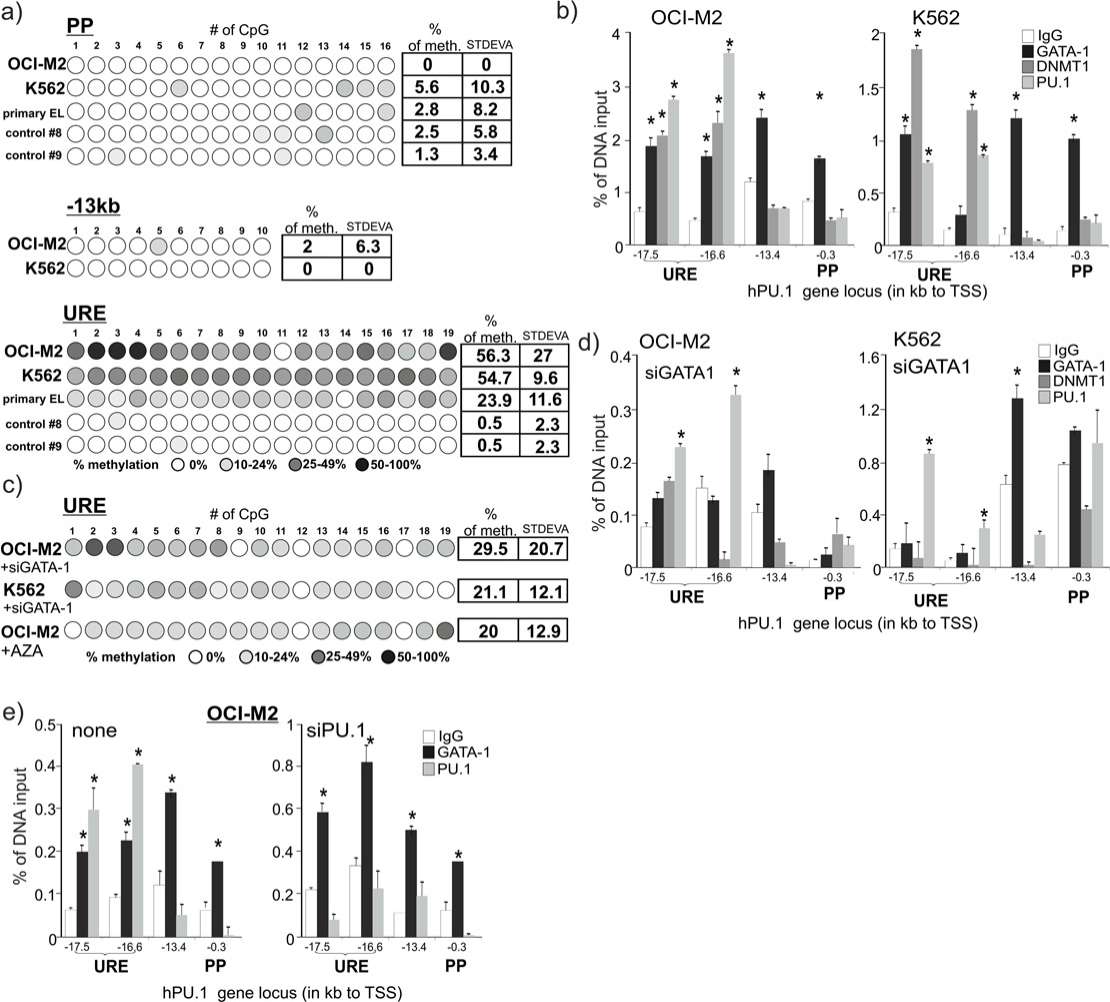
DNA methylation at PU.1 regulatory elements and DNMT1 occupancy in AML-ELs. DNA methylation analysis at the *PU*.*1* gene (a). Each circle corresponds to a CpG. Color shading indicates % methylation of PP, -13kb and URE in OCI-M2, K562, CD34+ cells isolated from bone marrow of AML-EL patient (primary EL) and normal bone marrow CD34+ (control #8 and #9). Tables show average DNA methylation in the indicated amplicons. (b) ChIP for GATA-1, DNMT1 and PU.1 occupancy at the *PU*.*1* gene in AML-ELs. Amplicon positions are shown on the X-axis. Specific signals are expressed as % of DNA input. Nonspecific signals of IgG immunoprecipitates: gray columns. Two independent experiments were done in duplicates. Error bars: SE, *p≤0.05. (c) DNA methylation at the URE in OCI-M2 and K562 after GATA-1 siRNA treatment and of OCI-M2 cells following treatment with 5uM AZA (both for 48hrs). (d) ChIP for GATA-1, DNMT1 and PU.1 occupancy at the *PU*.*1* gene 48hrs after GATA-1 siRNA or scrambled control oligo treatment in AML-ELs. (e) ChIP for GATA-1 and PU.1 occupancy at the *PU*.*1* gene 48hrs after PU.1 siRNA or scrambled control oligo treatment in OCI-M2.

We observed in hAML-ELs that repressive chromatin marks detected within the URE are targeted by GATA-1 and may mediate transcriptional inhibition of the *PU*.*1* gene. However, histone modifications are often considered to be temporary and relatively unstable chromatin marks compared to DNA methylation. Our previous data associated DNA methylation of the URE and PU.1 expression with responsiveness to the DNMT1-inhibitor 5’azacitidine (AZA) [21]. We therefore tested whether the DNA regions affected by GATA-1 in the AML-ELs are methylated. We also analyzed DNA methylation of URE and PP in the primary AML-M6 bone marrow CD34+ cells and in normal bone marrow CD34+ cells. The URE in two independent samples of normal CD34+ cells was not methylated. In contrast, the URE (in both primary and transformed ELs) was methylated while the -13.4E or PP regions were not (Fig 6A). This indicated that in AML-EL cells the GATA-1 (or PU.1) cooperates with a DNA methyl-transferase at the URE to transcriptionally repress the *PU*.*1* gene. As DNMT1 is the only DNA methyltransferase responsive to AZA, we decided to test the occupancy of DNMT1 on the *PU*.*1* gene in the AML-EL. Indeed, we detected significant DNMT1 occupancy of the URE but not the -13.4E or PP (Fig 6B), suggesting again that it is the URE that mediates the repressive activity imposed by GATA-1, which involves DNA methylation. PU.1 derepression resulting from the inhibition of GATA-1 by siRNA (or AZA treatment) was significantly revealed and it was accompanied by strong DNA demethylation at the URE (Fig 6C). To test whether GATA-1 recruits DNMT1 to URE, we inhibited GATA-1 in both hAML-ELs using siRNA and observed that indeed GATA-1 together with DNMT1 was expelled from the URE, whereas PU.1 binding remained unchanged (Fig 6D). Next, GATA-1 interaction with DNMT1 was tested using co-IP in both AML-ELs. We clearly observed a signal of GATA-1 in the precipitates generated with the anti-DNMT1 antibody. Unlike with GATA-1, we have not detected any PU.1 signal in the same precipitates suggesting that GATA-1, but not PU.1, can recruit DNMT1 in AML-EL (S9 Fig) and that GATA-1, rather than PU.1, is recruiting DNMT1 to the PU.1-bound-URE. To investigate whether the GATA1 binding to the URE is PU.1 dependent we performed ChIP for GATA-1 and PU.1 at the *PU*.*1* gene in AML-EL in which we utilized PU.1 siRNA or scrambled oligo. While PU.1 knockdown markedly decreased the PU.1 occupancy at the URE the GATA-1 occupancy remained stable at both the URE as well as at the PP (Fig 6E). Taken together, we have demonstrated that GATA-1 represses the *PU*.*1* gene by recruiting DNMT1, a process that can be reversed by AZA. In addition, GATA-1/DNMT1 interaction was revealed by Co-IP and DNA-localized to the URE by qChIP independently on PU.1 binding. We also observed that GATA-1-mediated repression in AML-EL regulates both the repressive histone modification and DNA methylation at region/s that are critical for inducing expression levels of the key myeloid transcription factor PU.1.

## Discussion

We herein described how lineage-specific transcription factor GATA-1 inhibits *PU*.*1* gene expression in human AML-EL cells and identified an as-yet unknown mechanism involving DNMT1, which co-occupies with GATA-1 at the PU.1 locus, specifically, at the URE that regulates most of the PU.1 expression. GATA-1 occupancy is a prerequisite of the *PU*.*1* gene repression at the URE independently on PU.1 association with the URE. Whereas deletion of URE decreases PU.1 to 20% and results in AML, further downregulation upon loss of p53 leads to even more aggressive AML [1, 28]. Unlike these mechanisms involved in myelomonocytic AML, we present mechanism that is involved likely at the earlier common progenitor level with a potential to produce erythroid as well as myeloid progeny. A relatively rare human AML-EL, exemplified by the two cell lines used here, have allowed us to clarify how bi-lineage blockade observed in ELs preserves the AML phenotype. This is also corroborated by analysis of the primary EL progenitors. The AML-EL display some similarities with normally differentiating erythroid progenitors where GATA-1 establishes lineage-specific repression of the myeloid program by repressing the *PU*.*1* gene completely. However, in ELs the co-expression of PU.1 and GATA-1 is maintained (S1 and S2 Figs).

We have observed several AML-EL-specific epigenetic alterations at the *PU*.*1* gene imposed by GATA-1 (Fig 4). GATA-1 has been shown to interact with several chromatin-remodeling and modification proteins [29, 30]. However this is likely to be the first report to document interactions with a DNA methyltransferase. GATA-1 is also capable to repress transcription by utilizing the Polycomb repressive complex, as shown at the *Hes1* gene in murine ELs [31] and the presence of H3K27Me at the *PU*.*1* gene supports this finding. The pattern of H3K27 hypermethylation at the URE in the human EL is also supported by another study [32]. GATA-1 negatively regulates a relatively large proportion of genes in mAML-EL and therefore it is likely that the repressive GATA-1-mediated mechanism observed at the *PU*.*1* gene is utilized also at other myeloid genes in hAML-ELs. Histone deacetylation has been a previously accepted mechanism for GATA-1-mediated inhibition of gene transcription as it interacts also with HDAC1, however more recent studies identified additional GATA-1 binding partners [30] within the MeCP1 complex that contain methyl-CpG-binding activity [33]. Thus, taken together, GATA-1 possesses the predicted chromatin remodeling functions through associations with partners that it can interact with.

Binding and repression of the *PU*.*1* gene by GATA-1 was also demonstrated in a recent publication that utilized murine embryonic stem cell-derived MPP-like cells isolated from GATA-1 -/- embryos [19]. Similarly to our data in human AML-ELs, the occupancy of GATA-1 (upon overexpression) was noted at the PU.1 promoter but much less PU.1 recruitment was noted at the URE [19] likely reflecting the interspecies differences [19]. Our data also support a role for GATA-1-mediated repression in the MEL cells (Fig 5). However, we also observed that the URE in MEL cells is strongly DNA-methylated and resistant to HMBA suggesting, that DNA demethylation of the URE is not required for MEL differentiation. Secondly, there are also other notable differences such as the *PU*.*1* gene in MEL cells not being enriched with H3K9Me3 or H3K27Me3 modification marks and instead the GATA-1-mediated repression is associated with a decline in H3K4Me3 modification (Fig 5). However, a decreasing trend in the H3K4Me3 marks at the PU.1 locus in mELs has an interesting correlate in hEL in that, upon treatment with AZA, the increase in H3K4Me3 observed at the URE is coincident with the PU.1 upregulation [21]. There is also the possibility that some histone modification changes across different cell lines could be explained by interspecies differences as, for example, is the case with avian myeloblasts which do not display any signs of the GATA-1-mediated suppression of the *PU*.*1* gene [18]. Our data demonstrate that in hAML-ELs there is a GATA-1-mediated repression mechanism operating through the PU.1 promoter (characterized by loss of active histone marks and accumulation of repressive histone marks). However, in addition to this we identified an additional AML-EL repression mechanism mediated through the URE that, in contrast to the promoter-mediated mechanism, also contains a DNA methylation mark. This is also supported by data from human AML-EL progenitors that do not show methylation at the promoter but instead are methylated in the URE similarly to the tested transformed AML-EL cell lines (Fig 6). As we also show that normal bone marrow CD34+ cells do not display any DNA methylation at the URE we consider the URE methylation to be an important pathophysiological mechanism of erythroid blockade in hAML-ELs and also of some higher risk MDS [21].

The transcriptional regulation of PU.1 involves not only the promoter but also the URE and additional three elements [20] that may become targeted by different regulators guiding spatial aspects of *PU*.*1* gene regulation [34]. Examples of these regulators include CTCF and Cohesin complex that are not only mutated in AML [35] but also may regulate PU.1 looping between URE and the promoter [36]. DNA methylation of the URE, herein presented, adds additional evidence of the importance of DNA methylation in leukemogenesis. Inhibition of DNA methylation has been shown to inhibit leukemic growth in patients [37]. GATA-1-mediated repression of the *PU*.*1* gene contrasts with other mechanisms that all represent positive transcriptional regulation of PU.1 involving RUNX1, ETV6, GATA-2, or C/EBPA (that are frequently mutated in hAML) [35]. Blocking the GATA-1/DNMT1-mediated repression by a DNA hypomethylating agent in hAML-EL (Fig 6) leads to a differentiation and proliferation arrest [21].

**Fig 7:**
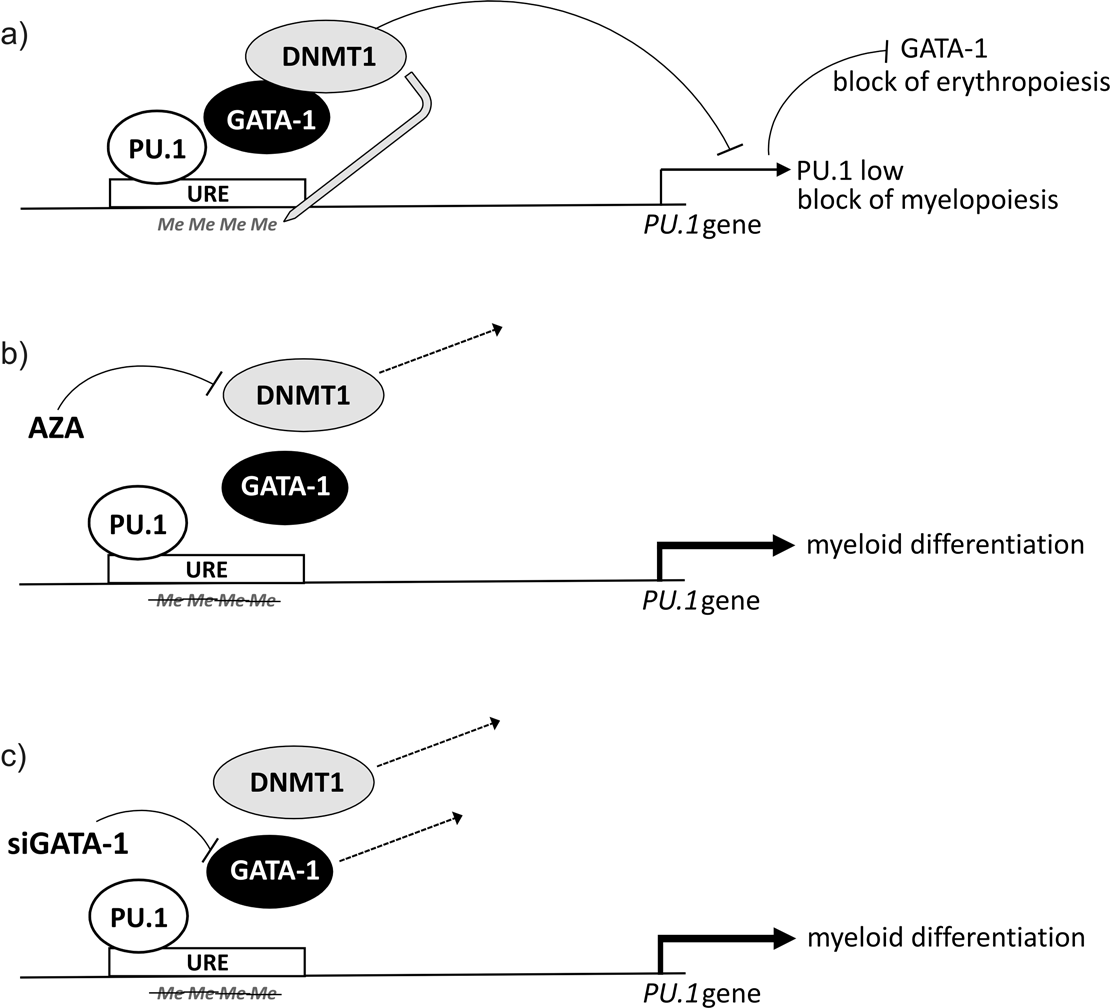
Model of GATA-1 mediated repression of the PU.1 gene in human EL. (a) GATA-1 recruits DNMT1 to URE leading to repressive DNA methylation at URE (abbrev. Me). This induces the *PU*.*1* gene repression and insufficient myelopoiesis in spite of the PU.1 independent autostimulation (by PU.1 protein binding to URE). (b) AZA mediated DNMT1 depletion from URE leads to DNA demethylation at the URE and stimulation of myelopoiesis through increasing PU.1 gene transcription. (c) Inhibition of GATA-1 in EL leads to DNMT1 depletion from URE, DNA demethylation and stimulation of myelopoiesis via PU.1 gene activation.

Data in our manuscript together with other data allowed us to generalize some of the conclusions. Briefly, we know that genetic loss of the URE leads to a decrease of the PU.1 level to 20% and results in AML in mouse [1] and its further downregulation (to 10%) resulted in a more aggressive AML [28]. In contrast, a full transcriptional repression of PU.1 expression blocks PU.1-dependent myelopoiesis and leads to the loss of the PU.1-dependent repression of GATA-1 targets, so it in turn facilitates the erythroid differentiation. In this manuscript we present evidence that methylation of the URE is also involved in downregulating PU.1 and associates with the AML-EL characterized by the incomplete repression of both myeloid and erythroid genes during differentiation. The methylation mark at the URE is fully reversible by either GATA-1 downregulation or DNMT1 inhibition with AZA (Fig 7). These observations open new avenues in rethinking the capabilities of differentiation therapy of AML-EL (including AZA), which would restore the control of cell proliferation, whilst stimulating hematopoietic differentiation.

## Supporting Information

S1 Appendix Relevant DNA sequences, primers, ChIP-Seq details. (DOCX)

S1 Fig GATA-1 and PU.1 are co-expressed in human AML-ELs. (a) GATA-1 (left graph) and PU.1 (right) expression in OCI-M2 (gray bars) and K562 cells (dark bars). HeLa cells served as control. White bars show expression in CD34+ bone marrow cells from EL patient. Expression data were normalized to HPRT mRNA. (b) Immunoblotting of GATA-1 and PU.1 in OCI-M2, K562 and HeLa cells; -beta- actin served as control.
(PDF)

S2 Fig Nuclear localization of GATA-1 and PU.1 in OCI-M2, K562 and HeLa cells. (a) Immunofluorescence for GATA-1 and PU.1 in OCI-M2, K562 and HeLa cells using confocal microscopy. DAPI is shown in the left panels. White bar: 5μm. (b) Merge of anti-PU.1, GATA-1 (left) and anti-PU.1, GATA-1, DAPI staining (right). Plots on the right show relative intensity and merge of fluorescence signals alongside white line depicted in the dark boxes.
(PDF)

S3 Fig Ectopic overexpression of GATA-1 upregulates PU.1 and its target genes in human AML-ELs. OCI-M2 (a) and K562 (b) cells were transfected with pXM-GATA-1 and cultured for 24hrs. Total mRNA was purified and subject to qRT-PCR (TaqMan). Y-axis: expression level of mRNAs (listed on X axis) relative to control samples transfected with pEEB-empty vector. Data were normalized to housekeeping HPRT gene expression. Star indicates t-test significance bellow 0.05. (c): Cell numbers determined for OCI-M2, K562 and HeLa cells upon GATA.1 overexpression up to 72hrs.
(PDF)

S4 Fig Extension of Fig 2. Additional PCR amplicons within PU.1 gene regulatory sites added. GATA-1 occupies the *PU*.*1* gene locus in AML-ELs. GATA-1 ChIP in OCI-M2 (left) and K562 (right). PCR amplicons positions are relative to TSS (kb). Specific signals are expressed as % of DNA input. Nonspecific signals of IgG immunoprecipitates are shown as gray columns. Two independent experiments were carried out in duplicate. Error bars: SE, *p≤0.05. Bottom: Vista plot of the *PU*.*1* gene with indicated positions of PCR amplicons. URE (upstream regulatory element), PP (proximal promoter region).
(PDF)

S5 Fig PU.1 occupancy at the *PU*.*1* gene locus. (a) Results from OCI-M2 and K562 (dark columns). Specific signals are expressed as % of DNA input. Nonspecific signals of IgG immunoprecipitates are shown as gray columns. X-axis: positions of PCR amplicons relative to TSS (kb). (b) PU.1, but not GATA-1, occupies the URE in the SKM-1 AML cells. ChIP data showing GATA-1 (gray) and PU.1 (dark) occupancies at the upstream regulatory regions of *PU*.*1* gene in SKM-1 (AML-M5) and HeLa cells. IgG control ChIP is shown as white columns. Error bars—SE of two independent experiments, *P ≤ 0.05.
(PDF)

S6 Fig Reporter gene assays ANOVA analysis- appendix to Fig 3. (a) ANOVA analysis between all relevant transfections (in table). Reporter gene assays showing that specific PU.1 element/s are repressed by GATA-1 in AML-ELs. The pGL3 basic plasmid was linked to the following upstream PU.1 elements: PP = proximal promoter, -12kbE, -14kbE, and the URE (or different combinations thereof, reporter constructs are named A-E were transfected into OCI-M2 and K562 cells either with scrambled control oligo (white bars) or with GATA-1 siRNA oligos (grey bars). HeLa cells served as control. Luciferase activity is normalized to the amount of proteins in each sample. (b) Reporter gene assays showing that the GATA-1 siRNA have no off target effects on the reporter constructs. The pGL3 basic plasmid was linked to the following upstream PU.1 elements: PP = proximal promoter and the URE were transfected into SKM1cells (AML-M5) either with scrambled control oligo (white bars) or with GATA-1 siRNA oligos (grey bars). Luciferase activity is normalized to the amount of proteins in each sample. Marks of significance: *p < 0.05, **p < 0.01, ***p < 0.001.
(PDF)

S7 Fig Histone modifications following GATA-1 overexpression in selected cell lines including human ELs. (a) SKM1 (left) and HeLa (right) cells. ChIP assay was used to detect H3K9Ac (gray bars) and H3K9Me3 (black bars) alongside upstream regulatory regions of the *PU*.*1* gene. Nonspecific signal is shown by white bars. (b) Extension of Fig 4 with additional PCR amplicons. Repressive histone modifications following GATA-1 overexpression in AML-ELs. ChIP at the *PU*.*1* gene locus was carried out for the H3K9Me3, H3K27Me3, and H3K9Ac histone tail modifications in OCI-M2 (left) and K562 (right) cells. Grey bars: control cells, dark bars: 48hrs after GATA-1 transgene transfection. Data are relative to control antibody IPs (Y axis). T-test significance: p<0.05 (star). Amplicon positions are shown on the X axis.
(PDF)

S8 Fig ChIP-seq read density profiles of H3K4me3, H3K9Ac, H3K9Me3 and H3K27Me3 datasets at the *PU*.*1* (Sfpi1) gene locus. Results from in MEL and MEL+DMSO cells (MEL induced to differentiate by 2% DMSO for 5 days; DNA sequence: -20kb upstream to 6kb downstream of PU.1 TSS). The proximal promoter (PP, 0 to 500nt upstream the TSS) and the URE (-13548bp; -14505bp) highlighted in vertical stripes.
(PDF)

S9 Fig co-IP immunoblot detection of GATA-1 and PU.1 in DNMT1 immunoprecipitates in OCI-M2 and K562 cells. Caption on the right indicate secondary Ab, caption below indicate specificity of immunoprecipitated DNA. Input protein lysates and equally loaded IgG control lines are also shown.
(PDF)
