# Supplementary material for: GATA-1 Inhibits PU.1 Gene via DNA and Histone H3K9 Methylation of Its Distal Enhancer in Erythroleukemia
Source: PLoS One. 2016 Mar 24;11(3):e0152234. doi: 10.1371/journal.pone.0152234 (PMC4807078; doi:10.1371/journal.pone.0152234)
Supplement: S1 Appendix — (DOCX) [file pone.0152234.s001.docx]

**Supplemental material**:

1) Human DNA sequences used in the reporter gene constructs:

URE: gccaattaagaggaaactgaggccaagtggagaggtgccagcccggggagtgcagcgaggagggctgttgggtgtccaggggcgggtgtctctgggtagatgggggtacctaggcctgaagagagatctgggtactggccagggaggcaggaggaaggagggaaggccgcggggcacccgggggctgctgcctggcaggtccccatgcccaggcaagggaagtttgttatttctcttgcttcgacttccccctttgatttattatagccatgaaatgctctgctctcttctcttttccttgctgtccctggggctggaggagcacgggcctccccgggagtgggcttcagcctccctagactcctgtctccttccaagggctaggcctgggggaccagaagcaagaggtgagtgacggccggcagggtgggaggaggagggcccatgcctccccaaggcaaggtggggcaggacccccagggaccagcacggtcccggttgggaggggctggggcccggcagagggtgtggcaggtgtggacgtggcaacaggcggctcccgggggtctcgggggatggcgggctctgggccaatggcctcagtgaggactgccagggtctgaaggttgggcacagagttcccggacgccaaacactaggtcagagaaatggcttttctgtgacccctgaccccacattctgatttaagggtggccaaagtaggcctggccctggctggcctggggattgagctgagagcccagaagaaggctgaggcctgaggcctgggggactctgggctcctccaggccgcggctggacatccccctgaggcctggcccaggctggcgagggccggaggctgtgtccggctcctcggcaggcctggtggccggagcgtttctctgggccgctgtgcggtgcctgtggtaatgggctgttggcgttttgcaatgggccgggggtggggaggcggcgcacacatgcttcctgtggtgactgggcgcttcctgttttctcaggcgccggccttgctgctgccgatgtggaaacaggggcagctgcagcccgggcggctccaggctgggcgctgtgaccctgcccagaggggcctacgtgggcccagcagccctaggccccagaccccagacccaagcagtggtggcccgagttttcccccacagcaggccaccatccctcccttccctaacagcttccgccaagaggaaggggcc

-14E: agtgctgagttggcggctcagcagctgctctggccacggaggcccagttataaataagcccagggtggccgagtcatcatgttctgaagctcccccagcccagctgcattacctcagtgaccacaggttgcaactgcccagaactgggcccccagaacccaccgacccaggcaaaggctggctgggttgcaggcatcctggggtcccccaatccagggcttgagcatctgtagctccttagatctgggcttccctggacacaggccacagaagggacaggcccatcctgccctacggagccgacagctatttacaaaccgaaaccaggagaagtgaagtggctgcggccaaagtggctccaggcccccagcccccacccccactccatccccagcagcccatccccctctccccttggcttcctctttccttccccgcacagctcccccacctcccactgcctggccagcccactctggattgacctgcaagcaccccatggtgtcccccaaccccacctctgcctggggctccccctctcctcttcaccccaggaccccatcagtctgagctcccgggagcaggccccctcccaccaggcccggcccaccctgcactacacaaggcccattcccacagttctcagttccccttcatgcttggaaacactccaaccccgatggtcacctccatgccaccggtcctcgcctgagggcagcttctcaggatgcccttgaccttcaagcagacacgagagggcccagaaatctggggtgctgtggtggagctggagcagctgaggccgtcagggggctcgtttct

-12E: tgtgaccagggcaggagcagggttggcctgaagctcaagggggaagcagggcgtggaccatagcaccacctgttaccttccctggctggccatcagcgcccagtggcccctgccgtctccccctgccctgccccaggggagggatcttcctgccatgttccttccctcctgaccagtcccaactgaggaggagcccggcctgagccccgctctgccgcaccagctggctgactgagcacccccgacctctctgggtcccacattcctcttctgcgaaattgtcattccacaaatacagagcgcctacttggtgctggccctgtgctgggcacaagctgtttctacagctgggacacagcccctgcccttgtggaacccatagtcctatggggaaggcagataacttacaaaatcctatgagctccaagagggaaaccaaggcagtgggacttagaggagttatgagtgttgcctggggtggggagaggtgttacttggatgggccttgaaggacagaaacgagtttgccagcaaaagcagagtggataggctccatgctgagtcacagccctgtgccttggctctagcatttcctctggtctgaagtgcctttctttggtctgggcctggtgaactcctattcatccatcagggcccatggacacaccatggcacaatcaggcctggcctggcctggtggctcctcaactccttcttcccgcccaaggcctgagcagacctggacgccaggcctcctttgtcacaataccctcaccagccccacccaggctggtgtctgctccctagctccagcccccgccgtcccctgctccgaaaagcaccgtttcccttttctgggtgaccacacgccatgcacagggaatttccatcatagcttgcggagcctctgattcactcgggcccctcacgcagttaactccttccaggcccaggcctcctgctccttcaccctgggctgccccccaacccctaaccgggacccctgggcatccctgcactgttcttgggccccagagcctcaaagctgaattcagctcctcattcactggcccctcagggcccactctcccttgtttgtcccaagggctcaaggcacacactcaggcagccttgggctaagagcacaggcttgagaggaattcaagcctgggtgcaaatcctgattttggcccttcttcctggagcaggtgtaagcgtccactctgagccccatttctcatctgtaaaatggataaccctattgtaaagatctcatgtgctgggcccatggtgggagctgtcattattagtttccttgtcttttcaaagggccctgctcatcttatcacagggtccctattccagagctaggccct

PP:

gaatggtggataggcaagaaaggtttgtaaaccaggagggttagattgatattcaggcattgccaagggatggggaaactgagaaacctgggctggaaacagagccctagggatttgaatgtggtaatcaagaagctgggaggggagggtataagaacccaggaccctcttctggtggtagggaagcccatgtgaacagattcagagggaaggaagaaagcagcactatgctgaagaccctacacctgagcccagcctcagcctagggatagcctaggcagtgggggtgcagggccacaggaagagccaggcctgatagcctgctagatcagagccctcacccaggaaggctgactccagaaagtggaggccccaagaggccaccccttccagctctgaccccaatgaggtgactgttggctacatagggatctcaaaagaagcaggcatttgttgggttagagcaaaagcctcccagtggtgtggcagagctacacgttctccctcccatcctccctccccagcttagcccccaaagtcatccctctcagtcccagcttcctctgggcagggggcctggaccccatggcttcccaggccagccctttgagcaccacctgccccagccggccagagacttcctgtatgtagcgcaagagatttatgcaaacgggttggggcggtgatgtcaccccaaggggactatctcccagcggcaggcccttcgataaaatcaggaacttgtgctggccctgcaatgtcaagggagggggctcacccagggctcctgtagctcagggggcaggcctgagccctgcacccgccccacgaccgtccagcccctgacggggcaccccatcctgagg

2) Primers used in qChIP analysis:

| GGATGGCTGAGGTTGATGGTTGA | hPU.1 (-17.5kb)-F |
| --- | --- |
| CAGCAGACAGGGATGAAGACAGAAGA | hPU.1 (-17.5kb)-R |
| CCTGACCCCACATTCTGATT | hPU.1 (-16.6kb)-F |
| CTTCTTCTGGGCTCTCAGC | hPU.1 (-16.6kb)-R |
| TGTGACCAGGGCAGGAGCAGG | hPU.1 (-13.4kb)-F |
| GCCAGCCAGGGAAGGTAACAGG | hPU.1 (-13.4kb)-R |
| tccctctcagtcccagcttcctc | hPU.1 (-0.3kb)-F |
| CCGTTTGCATAAATCTCTTGCGCTAC | hPU.1 (-0.3kb)-R |
| gaaacctgggctggaaacagagc | hPU.1 (-0.61kb)-F |
| GGCTTCCCTACCACCAGAAGAGG | hPU.1 (-0.61kb)-R |
| CCC TGT CGG GGT CAG TGC C HS2 gene -F  CAC ATT CTG TCT CAG GCA TCC HS2 gene- R |  |
|  |  |
|  |  |
|  |  |
|  |  |
|  |  |

3) Primers used in mRNA expression analysis (with TaqMan probe number):

| AGCCACATCGCTCAGACAC | hGAPDH-F #60 |
| --- | --- |
| GCCCAATACGACCAAATCC | hGAPDH-R #60 |
| TGACCTTGATTTATTTTGCATACC | hHPRT-F #73 |
| CGAGCAAGACGTTCAGTCCT | hHPRT-R #73 |
| ctgaggatattcagggacttgg | hCD14-F #87 |
| aactcttcggctgcctctt | hCD14-R #87 |
| cactgagcttgccacatcc | hGATA1-F #26 |
| atggagcctctggggatta | hGATA1- R#26 |
| ggagctgagatcccgaca | hCEBPA-F #28 |
| ttctaaggacaggcgtggag | hCEBPA-R #28 |
| CCACTGGAGGTGTCTGACG | hPU.1-F #27 |
| CTGGTACAGGCGGATCTTCT | hPU.1-R #27 |
| acccctggttcacctcct | hITGAM-F #36 |
| catgacataaggtcaaggctgt | hITGAM-R #36 |
|  |  |
|  |  |
|  |  |
|  |  |

4) Primers for detection of DNA methylation of the PU.1 gene:

PP Fwd GTTTTGTAATGTTAAGGGAGGG

PP Rev ACAACTACCCCTATTTCCACAT

-12E Fwd TGTGATTAGGGTAGGAGTAGGG

-12E Rev AAAAAAAACACTTCAAACCAAAA

URE Fwd GAGAAATGGTTTTTTTGTGATTT

URE Rev ACAACTACCCCTATTTCCACAT.

**5) ChIP-seq details**

Read density profiles used in the figures were aligned to the mm9 mouse reference genome using bowtie2 (PMID: 22388286) after merging the available replicates. The normalized signal files were generated using MACS1.4 (PMID: 22936215) with default parameters. For Encode datasets the aligned signal files (bigwig) were downloaded directly from the EncodeProject website ([https://www.encodeproject.org](https://www.encodeproject.org/)). Accession numbers and bibliographic references of all the datasets used are provided in table X.

Table of ChIP-seq datasets accession numbers:

| **Dataset** | **Accession #** | **Database** | **Reference (PMID)** |
| --- | --- | --- | --- |
| Gata1 Ter119+ | E-MTAB-1504 | ENA | 23519611 |
| Gata1 MEL | ENCFF001NSM | EncodeProject | 23193274 |
| Gata1 MELdmso | ENCFF001NSG | EncodeProject | 23193274 |
| PU1 EsEP | GSM545888/9 | GEO | 21695229 |
| PU1 MEL | GSM545878 | GEO | 21695229 |
| PU1 MacrLPS | GSM940927 | GEO | 23332752 |
| PU1 proB | GSM539537/8 | GEO | 22036565 |
| H3K4me3 MEL | ENCFF001MZR | EncodeProject | 23193274 |
| H3K4me3 MELdmso | ENCFF001KPU | EncodeProject | 23193274 |

6) The sequence of human URE:

distal URE: agaggaaactgaggccaagtggagaggtgccagcccggggagtgcagcgaggagggctgttgggtgtccaggggcgggtgtctctgggtagatgggggtacctaggcctgaagagagatctgggtactggccagggaggcaggaggaaggagggaaggccgcggggcacccgggggctgctgcctggcaggtccccatgcccaggcaagggaagtttgttatttctcttgcttcgacttccccctttgatttattatagccatgaaatgctctgctctcttctcttttccttgctgtccctggggctggaggagcacgggcctccccgggagtgggcttcagcctccctagactcctgtctccttccaagggctaggcctgggggaccagaagcaagaggtgagtgacggccggcagggtgggaggaggagggcccatgcctccccaaggcaaggtggggcaggacccccagggaccagcacggtcccggttgggaggggctggggcccggcagagggtgtggcaggtgtggacgtggcaacaggcggctcccgggggtctcgggggatggcgggctctgggccaatggcctcagtgaggactgcca

proximal URE: caatggcctcagtgaggactgccagggtctgaaggttgggcacagagttcccggacgccaaacactaggtcagagaaatggcttttctgtgacccctgaccccacattctgatttaagggtggccaaagtaggcctggccctggctggcctggggattgagctgagagcccagaagaaggctgaggcctgaggcctgggggactctgggctcctccaggccgcggctggacatccccctgaggcctggcccaggctggcgagggccggaggctgtgtccggctcctcggcaggcctggtggccggagcgtttctctgggccgctgtgcggtgcctgtggtaatgggctgttggcgttttgcaatgggccgggggtggggaggcggcgcacacatgcttcctgtggtgactgggcgcttcctgttttctcaggcgccggccttgctgctgccgatgtggaaacaggggcagctgcagcccgggcggctccaggctgggcgctgtgaccctgcccagaggggcctacgtgggcccagcagccctaggccccagaccccagacccaagcagtggtggcccgagttttcccccacagcaggccaccatccctcccttccctaacagcttccgccaaga
