# Supplementary figures and images for: GATA-1 Inhibits PU.1 Gene via DNA and Histone H3K9 Methylation of Its Distal Enhancer in Erythroleukemia
Source: PLoS One. 2016 Mar 24;11(3):e0152234. doi: 10.1371/journal.pone.0152234 (PMC4807078; doi:10.1371/journal.pone.0152234)

Figure S1

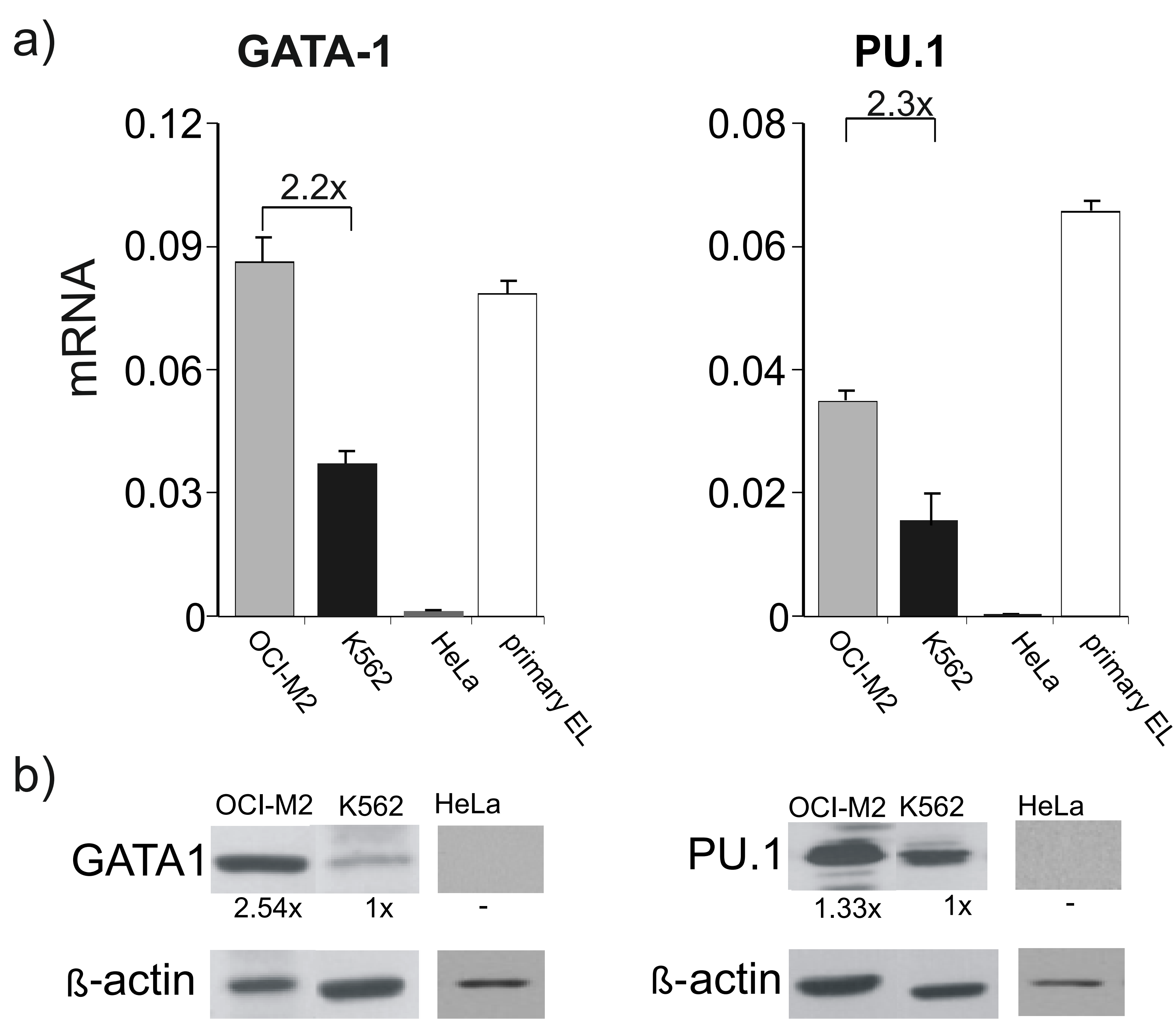

Supplement: S1 Fig — (a) GATA-1 (left graph) and PU.1 (right) expression in OCI-M2 (gray bars) and K562 cells (dark bars). HeLa cells served as control. White bars show expression in CD34+ bone marrow cells from EL patient. Expression data were normalized to HPRT mRNA. (b) Immunoblotting of GATA-1 and PU.1 in OCI-M2, K562 and HeLa cells; -beta- actin served as control. (PDF) [file pone.0152234.s002.pdf]

Figure S2

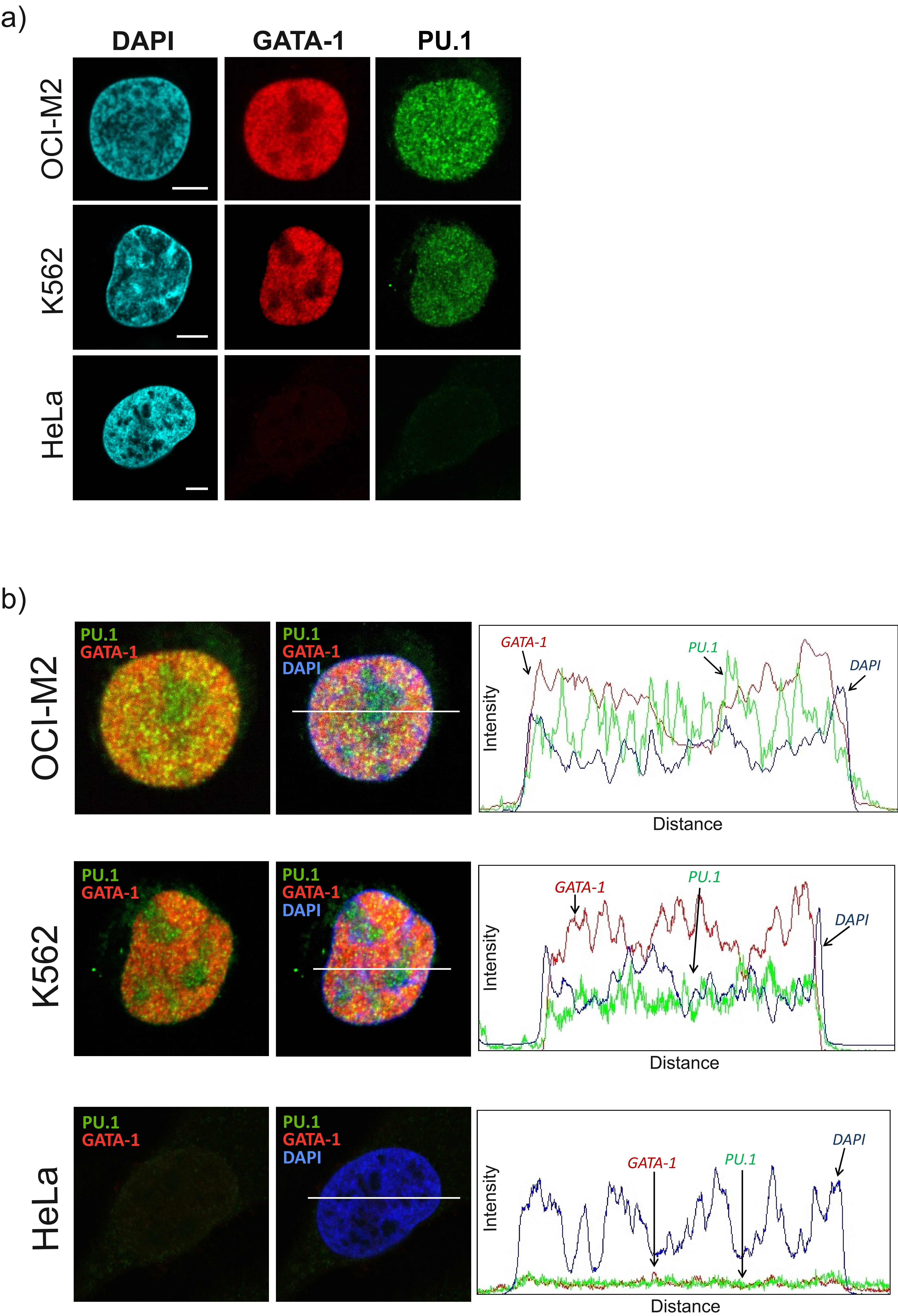

Supplement: S2 Fig — (a) Immunofluorescence for GATA-1 and PU.1 in OCI-M2, K562 and HeLa cells using confocal microscopy. DAPI is shown in the left panels. White bar: 5μm. (b) Merge of anti-PU.1, GATA-1 (left) and anti-PU.1, GATA-1, DAPI staining (right). Plots on the right show relative intensity and merge of fluorescence signals alongside white line depicted in the dark boxes. (PDF) [file pone.0152234.s003.pdf]

Figure S3

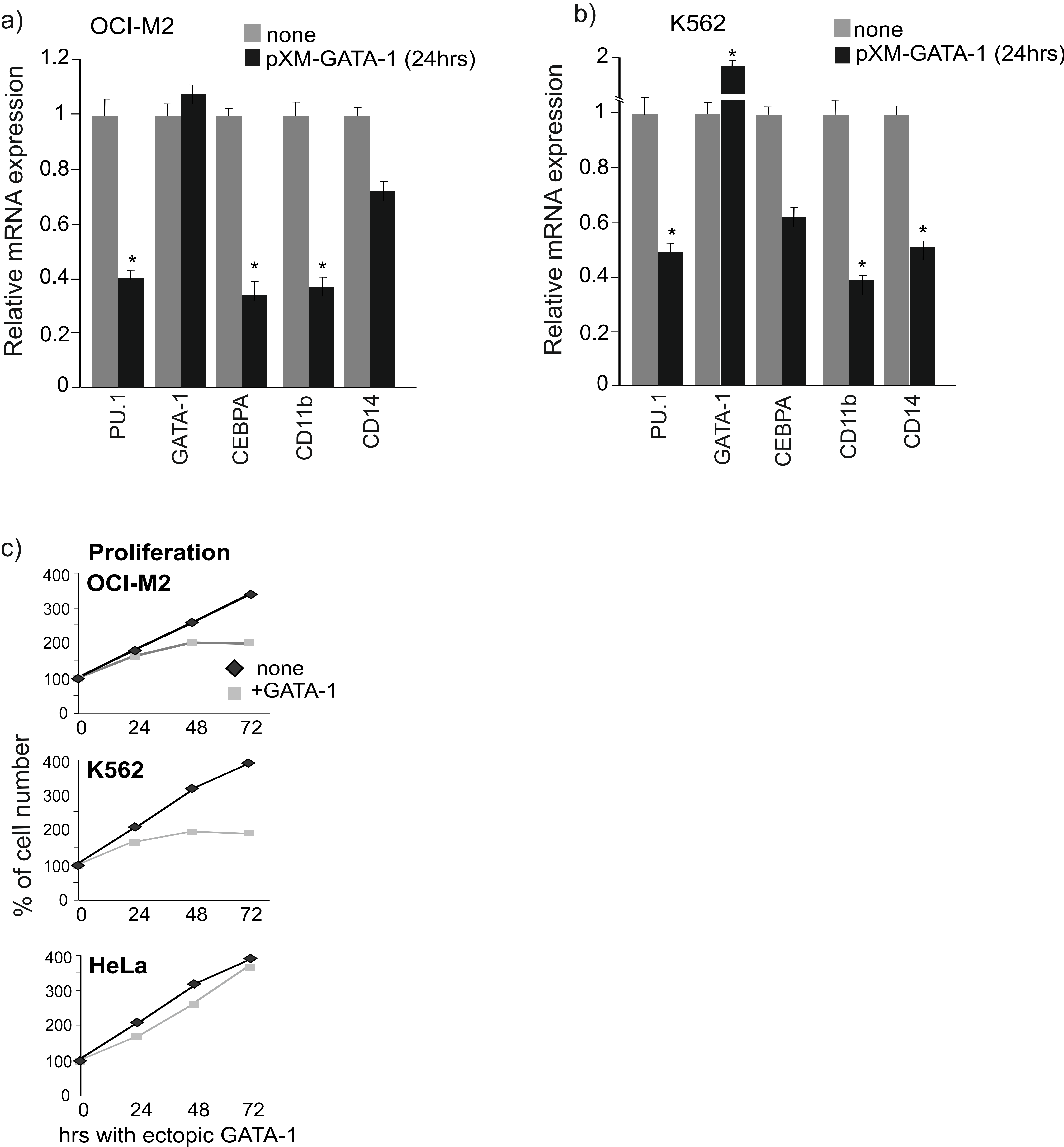

Supplement: S3 Fig — OCI-M2 (a) and K562 (b) cells were transfected with pXM-GATA-1 and cultured for 24hrs. Total mRNA was purified and subject to qRT-PCR (TaqMan). Y-axis: expression level of mRNAs (listed on X axis) relative to control samples transfected with pEEB-empty vector. Data were normalized to housekeeping HPRT gene expression. Star indicates t-test significance bellow 0.05. (c): Cell numbers determined for OCI-M2, K562 and HeLa cells upon GATA.1 overexpression up to 72hrs. (PDF) [file pone.0152234.s004.pdf]

Figure S4

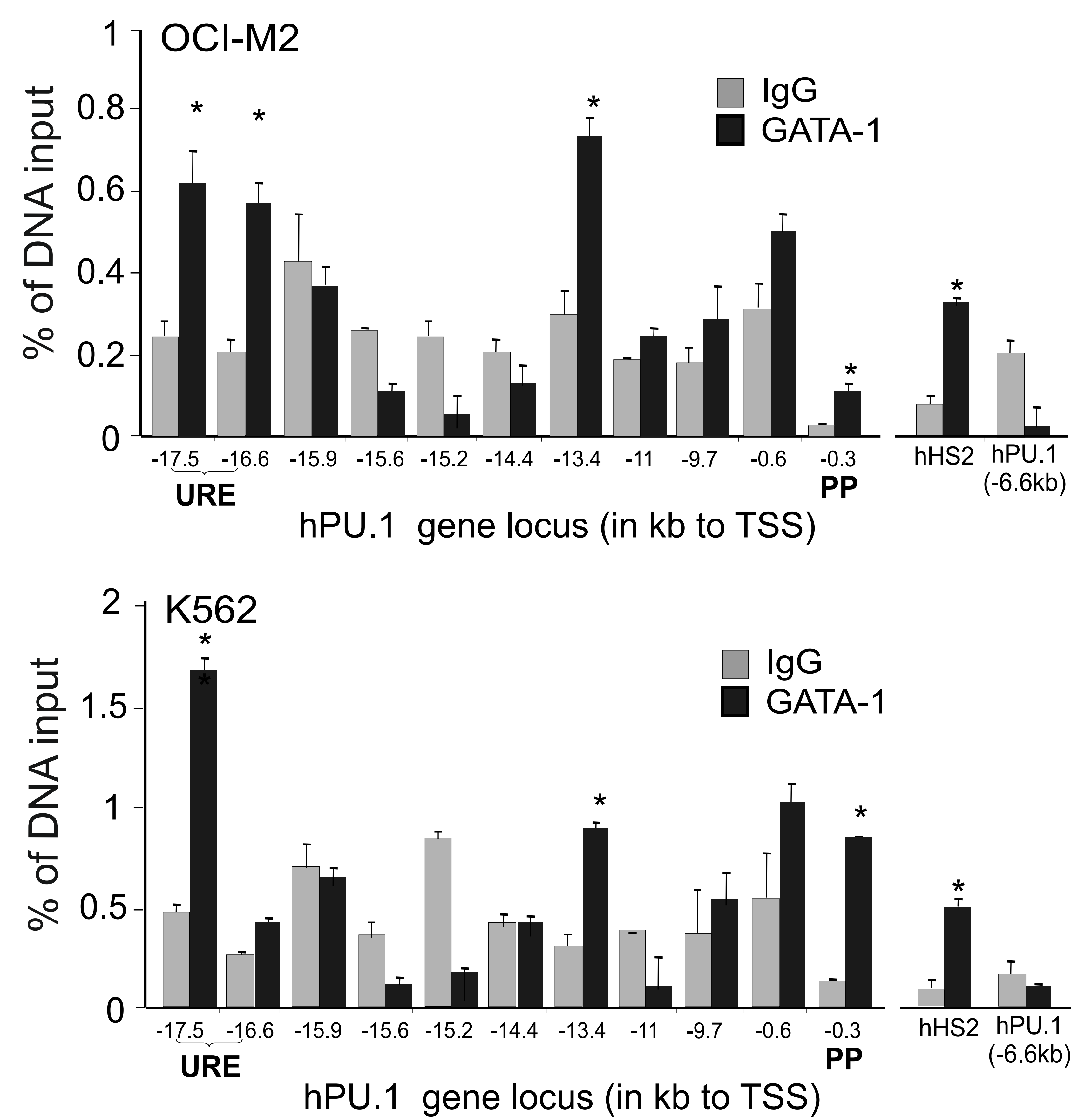

Supplement: S4 Fig — Additional PCR amplicons within PU.1 gene regulatory sites added. GATA-1 occupies the PU.1 gene locus in AML-ELs. GATA-1 ChIP in OCI-M2 (left) and K562 (right). PCR amplicons positions are relative to TSS (kb). Specific signals are expressed as % of DNA input. Nonspecific signals of IgG immunoprecipitates are shown as gray columns. Two independent experiments were carried out in duplicate. Error bars: SE, *p≤0.05. Bottom: Vista plot of the PU.1 gene with indicated positions of PCR amplicons. URE (upstream regulatory element), PP (proximal promoter region). (PDF) [file pone.0152234.s005.pdf]

Figure S5

a) **PU.1 ChIP**

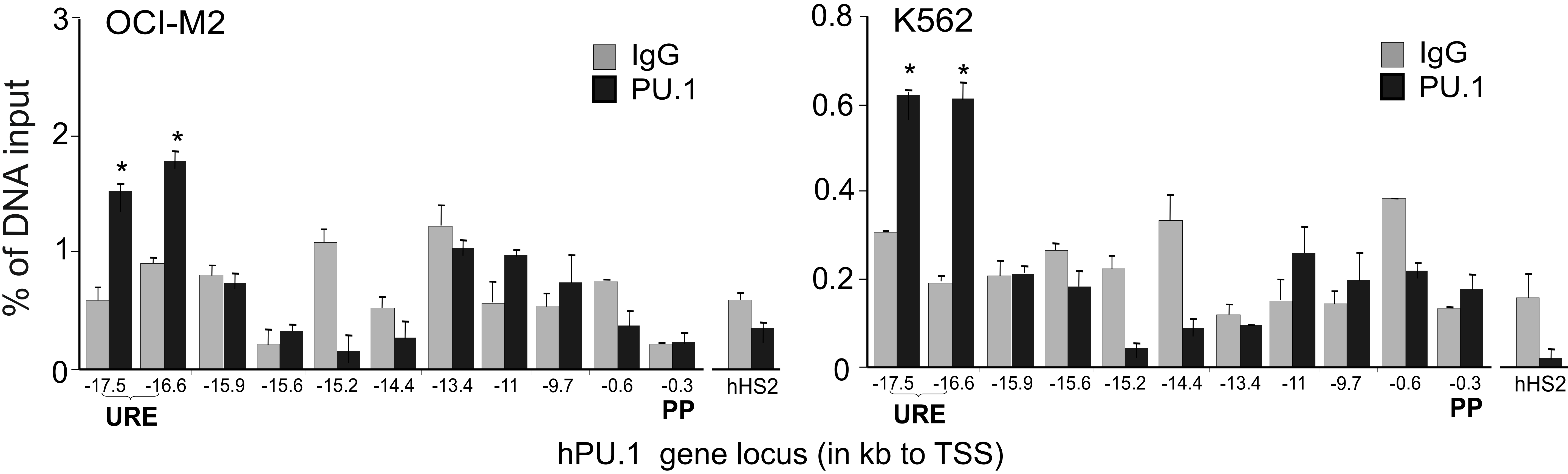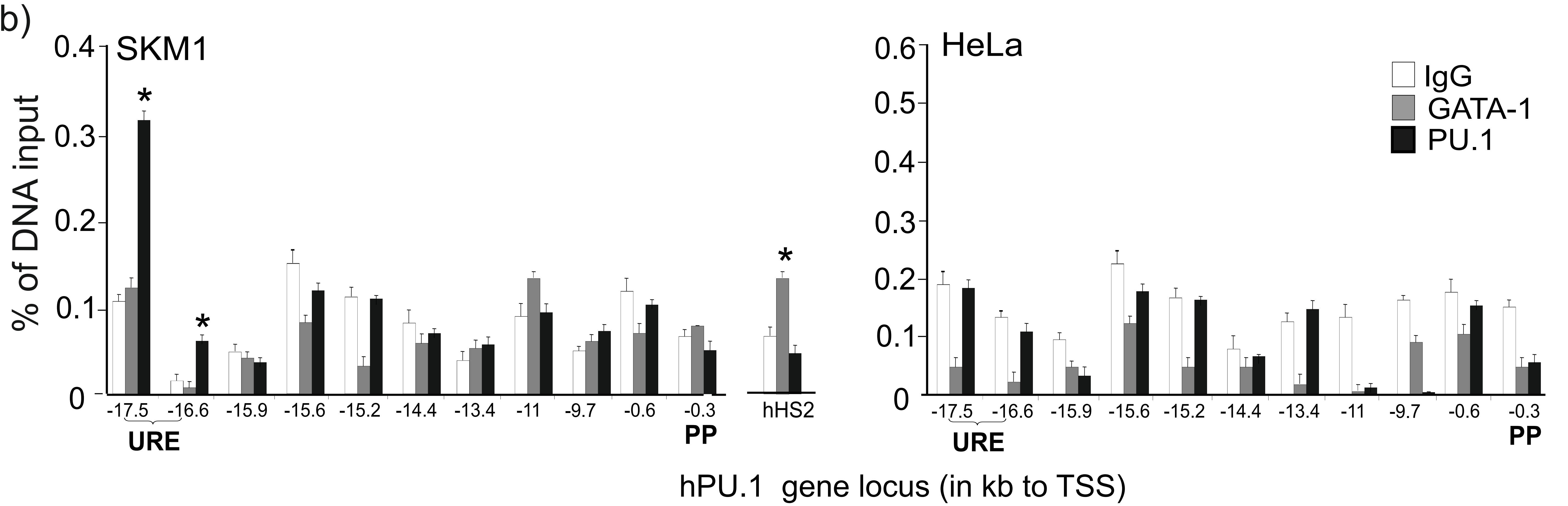

Supplement: S5 Fig — (a) Results from OCI-M2 and K562 (dark columns). Specific signals are expressed as % of DNA input. Nonspecific signals of IgG immunoprecipitates are shown as gray columns. X-axis: positions of PCR amplicons relative to TSS (kb). (b) PU.1, but not GATA-1, occupies the URE in the SKM-1 AML cells. ChIP data showing GATA-1 (gray) and PU.1 (dark) occupancies at the upstream regulatory regions of PU.1 gene in SKM-1 (AML-M5) and HeLa cells. IgG control ChIP is shown as white columns. Error bars—SE of two independent experiments, *P ≤ 0.05. (PDF) [file pone.0152234.s006.pdf]

Figure S7

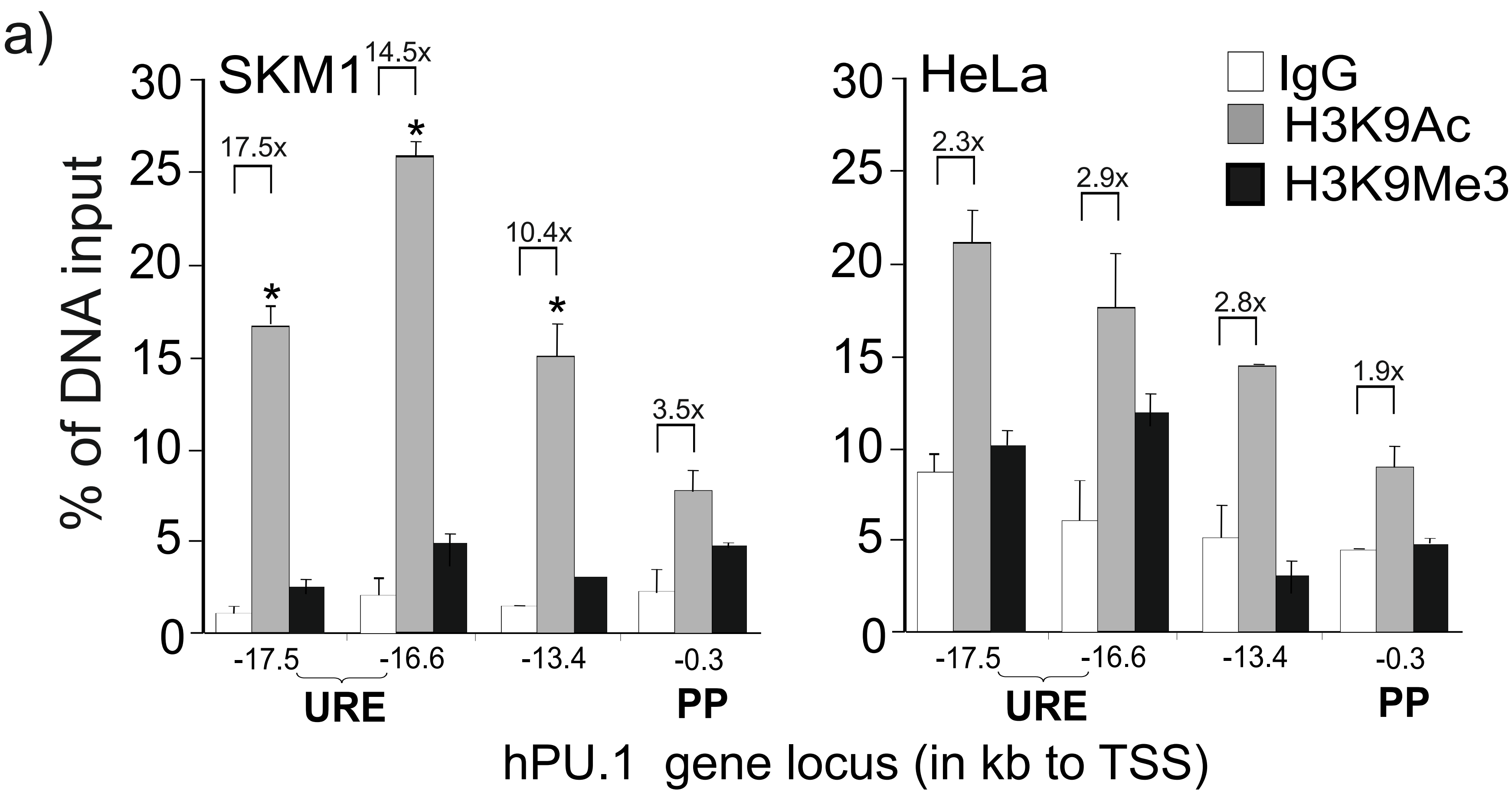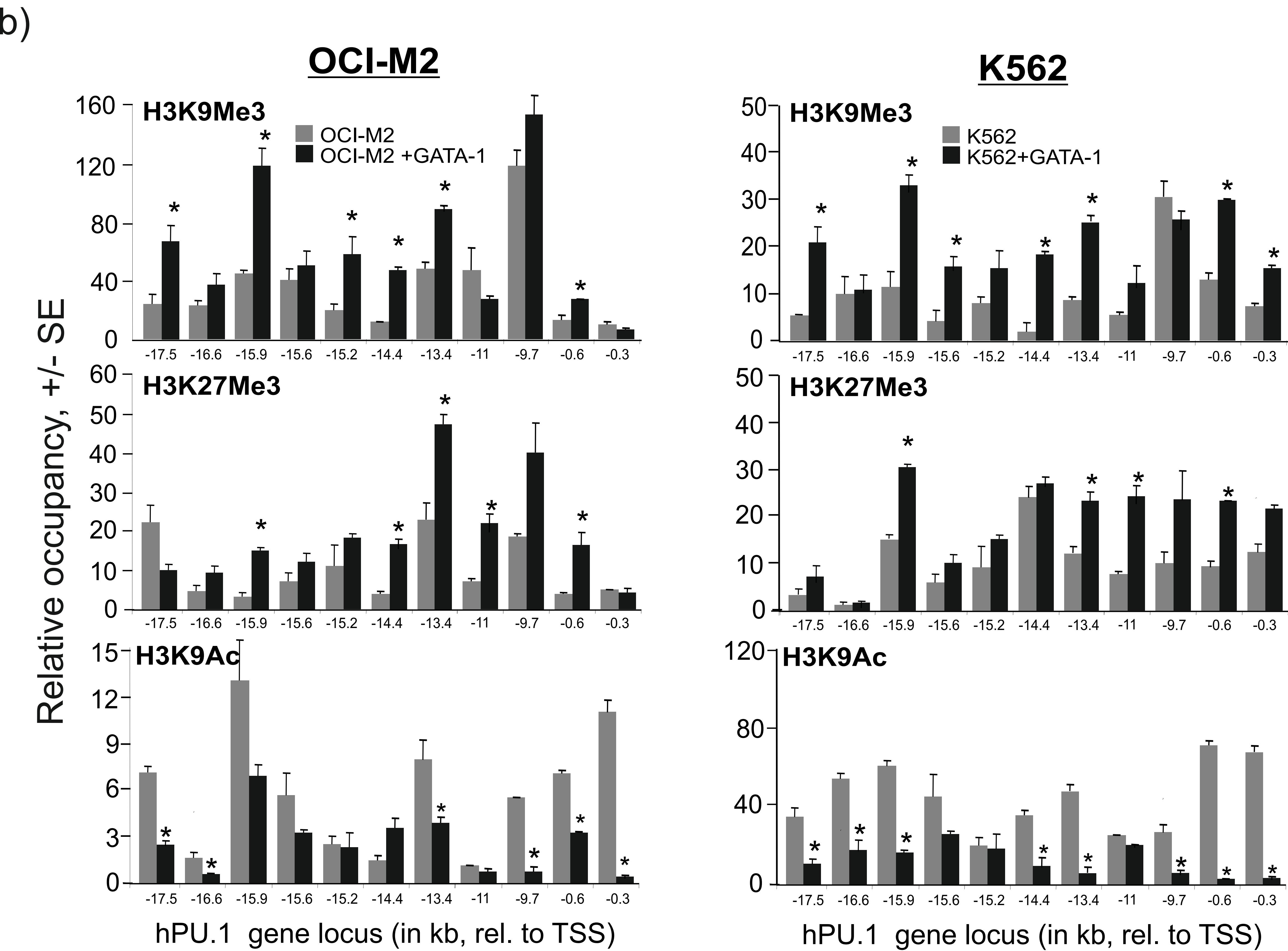

Supplement: S7 Fig — (a) SKM1 (left) and HeLa (right) cells. ChIP assay was used to detect H3K9Ac (gray bars) and H3K9Me3 (black bars) alongside upstream regulatory regions of the PU.1 gene. Nonspecific signal is shown by white bars. (b) Extension of Fig 4 with additional PCR amplicons. Repressive histone modifications following GATA-1 overexpression in AML-ELs. ChIP at the PU.1 gene locus was carried out for the H3K9Me3, H3K27Me3, and H3K9Ac histone tail modifications in OCI-M2 (left) and K562 (right) cells. Grey bars: control cells, dark bars: 48hrs after GATA-1 transgene transfection. Data are relative to control antibody IPs (Y axis). T-test significance: p<0.05 (star). Amplicon positions are shown on the X axis. (PDF) [file pone.0152234.s008.pdf]

Figure S8

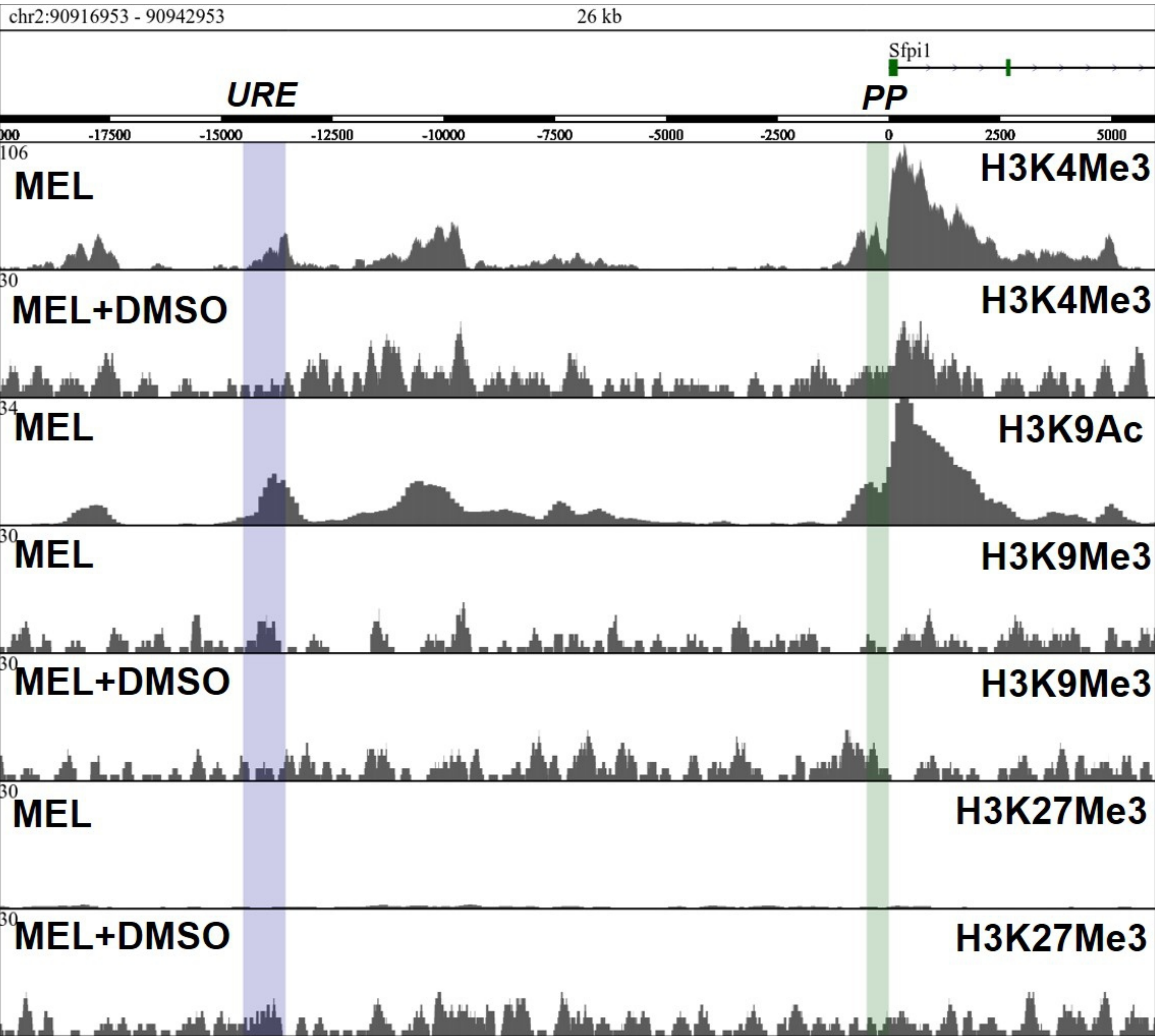

Supplement: S8 Fig — Results from in MEL and MEL+DMSO cells (MEL induced to differentiate by 2% DMSO for 5 days; DNA sequence: -20kb upstream to 6kb downstream of PU.1 TSS). The proximal promoter (PP, 0 to 500nt upstream the TSS) and the URE (-13548bp; -14505bp) highlighted in vertical stripes. (PDF) [file pone.0152234.s009.pdf]

Figure S9

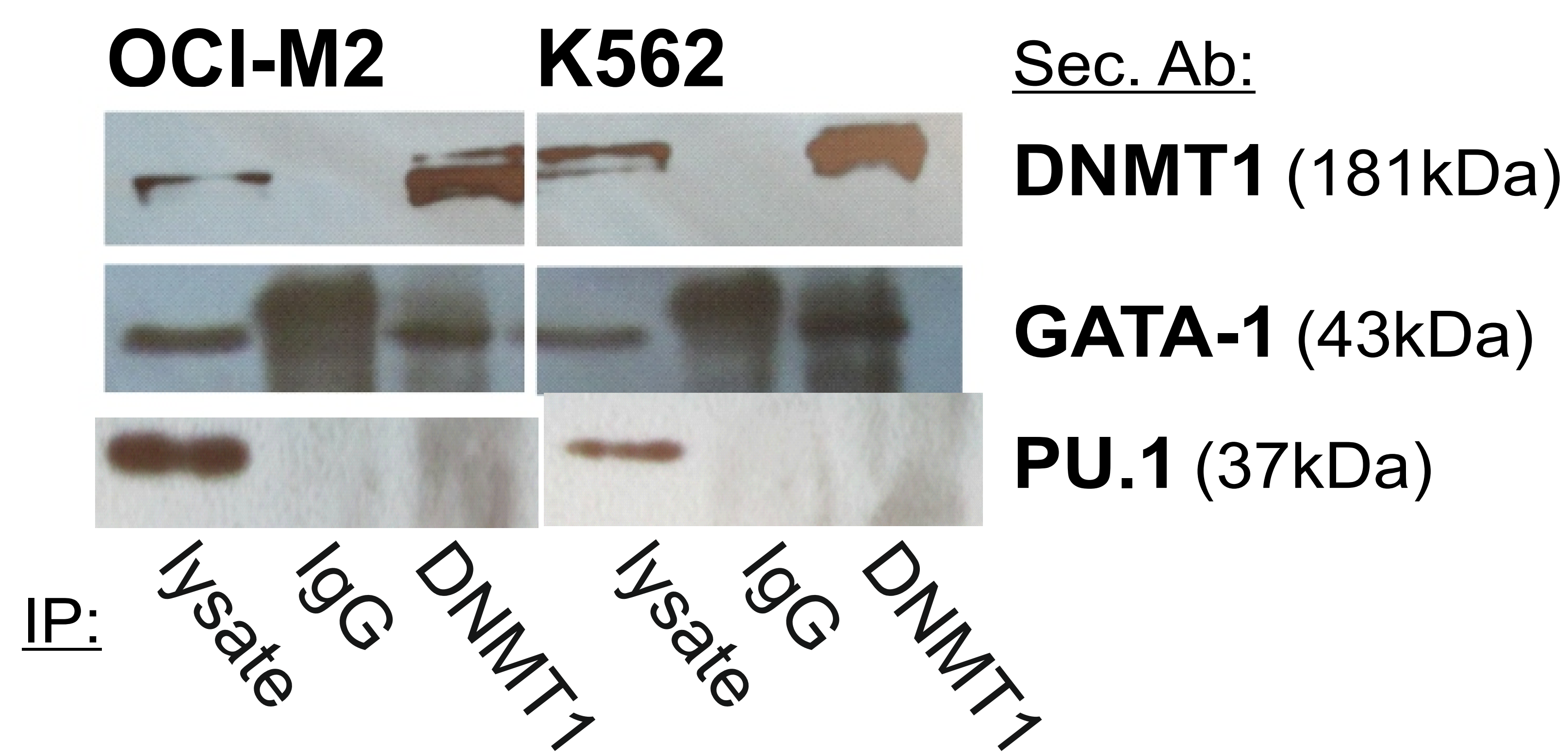

Supplement: S9 Fig — Caption on the right indicate secondary Ab, caption below indicate specificity of immunoprecipitated DNA. Input protein lysates and equally loaded IgG control lines are also shown. (PDF) [file pone.0152234.s010.pdf]
