# Supplementary material for: GATA-1 Inhibits PU.1 Gene via DNA and Histone H3K9 Methylation of Its Distal Enhancer in Erythroleukemia
Source: PLoS One. 2016 Mar 24;11(3):e0152234. doi: 10.1371/journal.pone.0152234 (PMC4807078; doi:10.1371/journal.pone.0152234)

Figure S6

a)

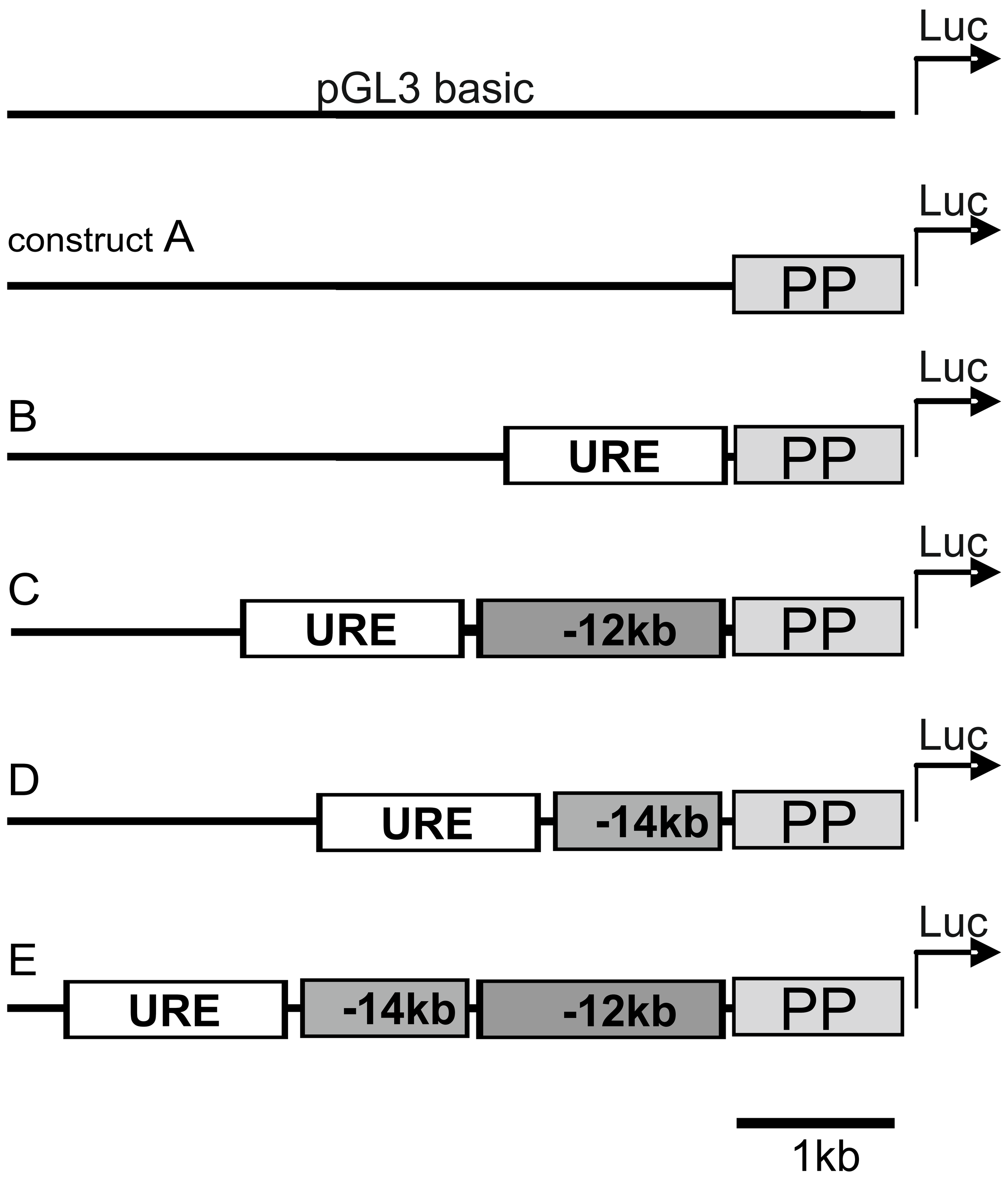

ANOVA analysis

	OCI-M2 Control	OCI-M2 siRNA	K562 Control	K562 siRNA
A vs B	**	***	**	***
A vs C	**	**	***	***
A vs D	**	***	***	***
A vs E	*	**	***	***
B vs C	NS	NS	NS	NS
B vs D	*	NS	NS	NS
B vs E	*	NS	NS	NS
C vs D	NS	NS	NS	NS
C vs E	NS	NS	NS	NS
D vs E	NS	NS	NS	NS

NS ...non significant

b)

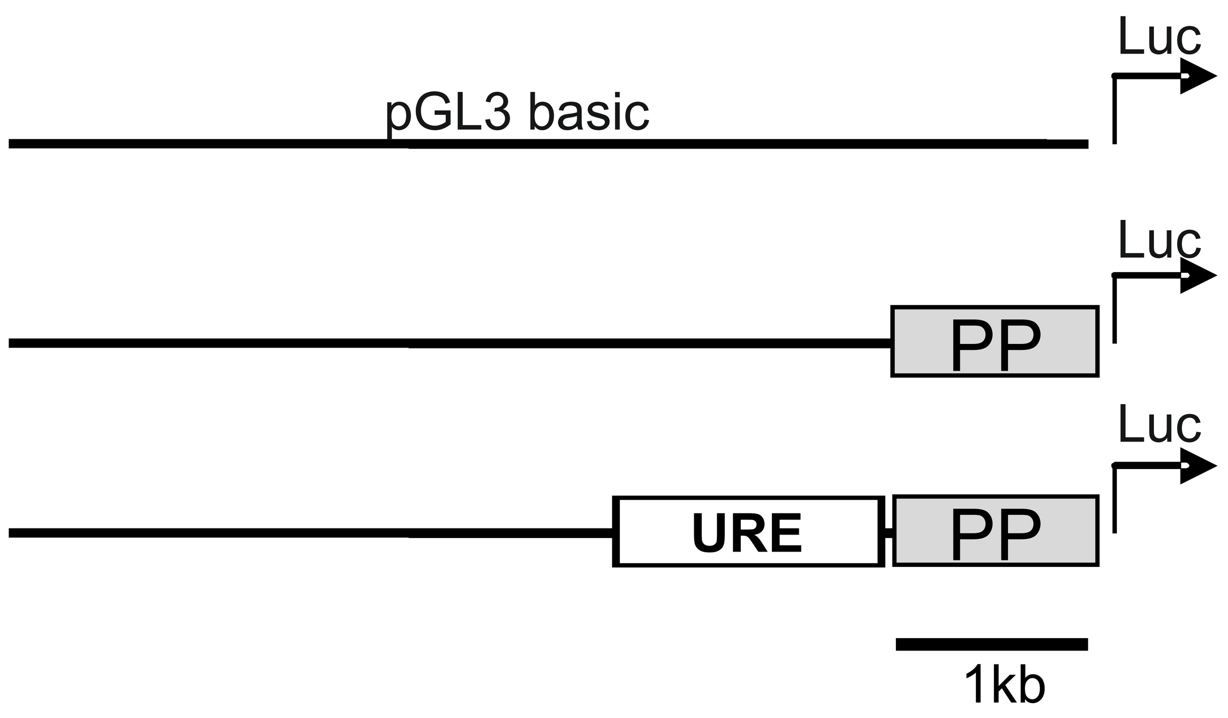

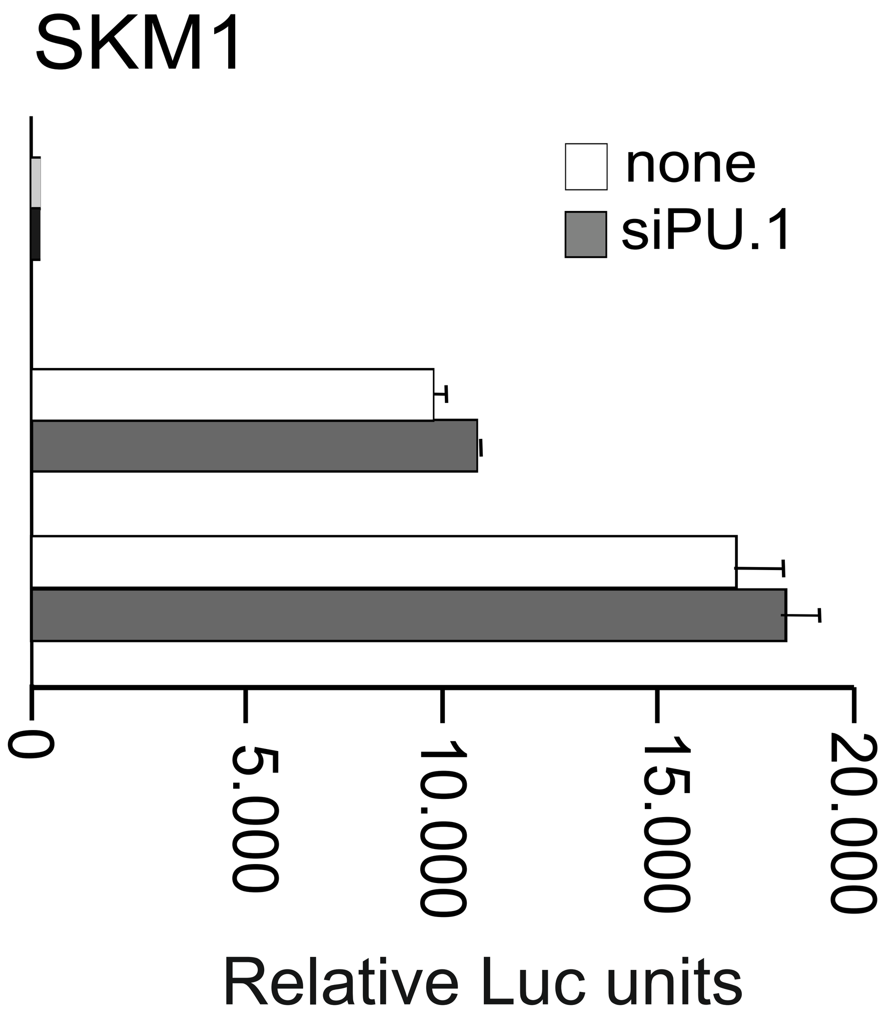

Supplement: S6 Fig — (a) ANOVA analysis between all relevant transfections (in table). Reporter gene assays showing that specific PU.1 element/s are repressed by GATA-1 in AML-ELs. The pGL3 basic plasmid was linked to the following upstream PU.1 elements: PP = proximal promoter, -12kbE, -14kbE, and the URE (or different combinations thereof, reporter constructs are named A-E were transfected into OCI-M2 and K562 cells either with scrambled control oligo (white bars) or with GATA-1 siRNA oligos (grey bars). HeLa cells served as control. Luciferase activity is normalized to the amount of proteins in each sample. (b) Reporter gene assays showing that the GATA-1 siRNA have no off target effects on the reporter constructs. The pGL3 basic plasmid was linked to the following upstream PU.1 elements: PP = proximal promoter and the URE were transfected into SKM1cells (AML-M5) either with scrambled control oligo (white bars) or with GATA-1 siRNA oligos (grey bars). Luciferase activity is normalized to the amount of proteins in each sample. Marks of significance: *p < 0.05, **p < 0.01, ***p < 0.001. (PDF) [file pone.0152234.s007.pdf]
